# A High-Throughput Clinical Laboratory Methodology for the Therapeutic Monitoring of Ibrutinib and Dihydrodiol Ibrutinib

**DOI:** 10.3390/molecules27154766

**Published:** 2022-07-25

**Authors:** Gellért Balázs Karvaly, István Vincze, Alexandra Balogh, Zoltán Köllő, Csaba Bödör, Barna Vásárhelyi

**Affiliations:** 1 Department of Laboratory Medicine, Semmelweis University, 4 Nagyvárad tér, 1089 Budapest, Hungary; vincze.istvan@pharma.semmelweis-univ.hu (I.V.); kollo.zoltan@med.semmelweis-univ.hu (Z.K.); vasarhelyi.barna@med.semmelweis-univ.hu (B.V.); 2 Department of Internal Medicine and Hematology, Semmelweis University, 46 Szentkirályi Utca, 1088 Budapest, Hungary; balogh.alexandra@med.semmelweis-univ.hu; 3 Department of Pathology and Experimental Cancer Research, Semmelweis University, 26 Üllői út, 1085 Budapest, Hungary; bodor.csaba1@med.semmelweis-univ.hu; 4 HCEMM-SE Molecular Oncohematology Research Group, 26 Üllői út, 1085 Budapest, Hungary

**Keywords:** tyrosine kinase inhibitor, liquid chromatography–mass spectrometry, active metabolite, therapeutic drug monitoring, chronic lymphocytic leukemia, assay error equation, oral anticancer drug

## Abstract

Ibrutinib (IBR) is an oral anticancer medication that inhibits Bruton tyrosine kinase irreversibly. Due to the high risk of adverse effects and its pharmacokinetic variability, the safe and effective use of IBR is expected to be facilitated by precision dosing. Delivering suitable clinical laboratory information on IBR is a prerequisite of constructing fit-for-purpose population and individual pharmacokinetic models. The validation of a dedicated high-throughput method using liquid chromatography–mass spectrometry is presented for the simultaneous analysis of IBR and its pharmacologically active metabolite dihydrodiol ibrutinib (DIB) in human plasma. The 6 h benchtop stability of IBR, DIB, and the active moiety (IBR + DIB) was assessed in whole blood and in plasma to identify any risk of degradation before samples reach the laboratory. In addition, four regression algorithms were tested to determine the optimal assay error equations of IBR, DIB, and the active moiety, which are essential for the correct estimation of the error of their future nonparametric pharmacokinetic models. The noncompartmental pharmacokinetic properties of IBR and the active moiety were evaluated in three patients diagnosed with chronic lymphocytic leukemia to provide a proof of concept. The presented methodology allows clinical laboratories to efficiently support pharmacokinetics-based precision pharmacotherapy with IBR.

## 1. Introduction

**Figure 1:**
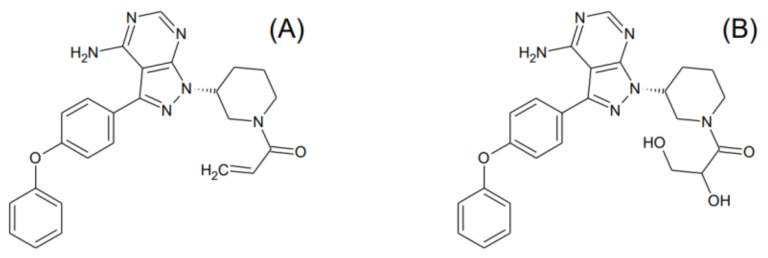
Structural formulae of (**A**) ibrutinib and (**B**) dihydrodiol ibrutinib.

Ibrutinib (1-[(3R)-3-[4-Amino-3-(4-phenoxyphenyl)-1H-pyrazolo[3,4-d]pyrimidin-1-yl]-1-piperidinyl]-2-propen-1-one, chemical abstracts service number 936563-96-1, IBR) is a first-in-class, small-molecule, nonpeptide, nonnucleobase oral anticancer drug. First approved in 2013, its pharmacological indications include the treatment of mantle-cell lymphoma, chronic lymphocytic leukemia with or without 17p deletion, and Waldenström’s macroglobulinemia. Its pharmacological action is exerted through the irreversible inhibition of Bruton tyrosine kinase, a signaling molecule of the B-cell antigen receptor and cytokine receptor pathways [1]. IBR is transformed extensively into a pharmacologically active metabolite dihydrodiol ibrutinib (1-[(3R)-3-[4-amino-3-(4-phenoxyphenyl)pyrazolo[3,4-d]pyrimidin-1-yl]piperidin-1-yl]-2,3-dihydroxypropan-1-one, chemical abstracts service number 1654820-87-7, DIB), with DIB/IBR concentration ratios of 1:1 to 3:1 being typically attained (Figure 1) [2]. IBR has been useful primarily in combination therapies (immunotherapy with obinutuzumab, ofatumumab, rituximab, or ublituximab; chemoimmunotherapy with fludarabine–cyclophosphamide–rituximab or bendamustine–rituximab; chimeric antigen receptor T-cell therapy; as well as concurrent treatment with the Bcl-2 protein inhibitor venetoclax, or with the phosphatidylinositol-3-kinase inhibitors duvelisib or idelalisib) [3].

Treatment with IBR requires careful guidance in dosing, primarily due to its severe adverse effects caused by off-target kinase inhibition and other mechanisms [4]. These include atrial fibrillation, the most common reason for the discontinuation of IBR therapy, major bleeding (occurring in 1–10% of patients), general debility, arthralgia, infection (especially pneumonitis), and secondary malignancy. Fatalities associated with these have been reported in up to 10%, while dose modification is prompted in 11–50% of cases [5]. A multicenter, retrospective chart study including adults treated with chronic lymphocytic leukemia revealed that—mainly due to the occurrence of adverse events—25% of patients experienced at least one dose reduction, while treatment discontinuation and dose holds impacted 20% and 34% of cases, respectively [6].

The poor solubility of IBR in water, its low permeability through membranes (Biopharmaceutics Classification System Class II), and extensive metabolism catalyzed by cytochrome P450 3A (CYP3A) results in considerable variability in its pharmacokinetic properties [7,8]. IBR has low oral bioavailability, high (>95%) affinity and special binding properties to plasma albumin, a large apparent volume of distribution, and changes in hepatic metabolism when coadministered with CYP3A inhibitors or inducers [8,9,10]. A population modeling study identified 67% interindividual and 47% intraindividual variability in the clearance of IBR, as well as 51% and 26% in the case of DIB, respectively. IBR exposure was higher in subjects with one copy of the CYP 3A4*22 variant. Nevertheless, when tested as candidate covariates, neither anthropometric or demographic properties of individuals, or the results of a wide range of laboratory tests, have proved to have a major impact on the pharmacokinetic behavior of IBR or DIB [10]. Recent discussions over IBR dose reduction and the clinical impact of related drug–drug and drug–food interactions have also highlighted the importance of individual therapy guidance [11,12,13,14].

The translation of therapeutic drug monitoring (TDM) results into clinically meaningful information using precision pharmacotherapy software is the most promising approach to addressing these issues and to optimizing the dosing regimens of IBR. Predicting IBR and DIB plasma concentrations using nonparametric pharmacokinetic modeling is a particularly attractive strategy, since the pharmacokinetic properties of IBR observed in each individual are retained instead of being melted into summary statistics [15,16,17]. The error of predictions, i.e., the differences between observed concentrations and those predicted by the model, is estimated as the combination of the measurable analytical error, derived from the standard deviation (SD) of each measurement result, and an unmeasurable “noise” of clinical and pharmaceutical origin [15,18]. Since the processing of each real-life TDM sample in several repeats is beyond clinical reality, the efficient estimation of the imprecision of measured IBR and DIB concentrations by applying empirical assay error equations is a key component of building their nonparametric pharmacokinetic models, and should be part of method validation [18,19]. Evidence shows that, concerning analysis relying on liquid chromatography–tandem mass spectrometry (LC-MS/MS) and the use of internal standards, the relationship between drug concentrations and SDs can be characterized with linear models [20,21,22]. Nonparametric population pharmacokinetic models incorporating linear assay error equations have been constructed for voriconazole, as well as for atorvastatin and its pharmacologically active metabolites [19,23].

An important prerequisite of reporting reliable assay results for efficient pharmacokinetic modeling is the evaluation of the stability of IBR and DIB in samples in the preanalytical and analytical phases. IBR has acceptable stability in heparinized plasma stored at 4 °C or lower, or when exposing the samples to multiple freeze–thaw cycles, but not at ambient temperature [24]. Rood et al. measured the concentrations of both IBR and DIB after keeping heparinized plasma samples at 0 °C for 2 h, or at −80 °C for 2 months, in addition to performing a freeze–thaw experiment and assessment of the autosampler stability of prepared samples [25]. However, no data have been published concerning IBR and DIB stability in whole blood or in plasma in the early preanalytical phase, i.e., before the samples reach the premises of the laboratory. In this early phase, patient samples are frequently kept on the bench at ambient temperature for an undefined length of time. Performing investigations for controlling for this phase is therefore pivotal.

Our aim is to present the results of experiments accomplished to attain comprehensive clinical laboratory information required for constructing nonparametric pharmacokinetic models that can be employed efficiently for individually optimized treatments with IBR. These experiments targeted (1) the development and validation of a high-throughput analytical method for the clinical analysis of IBR and DIB in human plasma, (2) the characterization of the stability of IBR, DIB, and the active moiety (represented by the sums of IBR and DIB concentrations) in the collected blood samples and in plasma separated in the early preanalytical phase, and (3) the construction of assay error equations of IBR, DIB, and the active moiety, which are incorporable into nonparametric pharmacokinetic models as the measurable error. A proof of concept of the developed methodology is provided by the evaluation of the pharmacokinetics of IBR, DIB, and the active moiety in three patients diagnosed with chronic lymphocytic leukemia.

## 2. Materials and Methods

### 2.1. Chemicals and Solutions

Ibrutinib (99%), dihydrodiol ibrutinib (99%), ^2^H_5_-ibrutinib and ^2^H_5_-dihydrodiol ibrutinib were purchased from Alsachim S.A.S. (Illkirch-Grafenstaden, France). LC-MS grade acetonitrile, formic acid, methanol and water were supplied by Reanal Labor (Budapest, Hungary).

Stock solutions (4 mg/mL) of the analytes, and 1 mg/mL stock solutions of the isotopically labeled internal standards (IS), respectively, were prepared in methanol. The solutions employed for spiking blank human plasma samples in the experiments conducted to establish the assay error equations contained IBR and DIB in the range of 0.011–26.1 µg/mL. The concentration of ^2^H_5_-IBR and ^2^H_5_-DIB in the IS working solution was 10 µg/mL. 1.4 µL IS working solution was added to each milliliter of acetonitrile employed for the deproteinization of plasma samples.

### 2.2. Sample Preparation

Deproteinizing solution (200 µL) was added to 50 µL plasma on a Phenomenex Impact 96-well protein precipitation plate (Gen-Lab, Budapest, Hungary). The plate was shaken at 1100 rpm for 10 min on an Allsheng TMS-200 thermoshaker incubator (Lab-Ex, Budapest, Hungary), and the supernatant was transferred to a collection plate (1 mL/slot). By applying nitrogen (purity rating 5.0) at gentle positive pressure using a Phenomenex Presston 100 positive-pressure manifold (Gen-Lab, Budapest, Hungary), the supernatant could be filtered successfully without carrying along solid particles. Further processing was therefore possible without centrifugation. Supernatant (150 µL) was mixed with 90 µL water, and the mixture was submitted for analysis.

### 2.3. Analysis

A modular CE-IVD certified liquid chromatograph–tandem mass spectrometer consisting of a Shimadzu DGU20 CL degasser, two LC30-AD CL pumps, a SIL-30-CL autosampler, a CTO-20AC column oven and an LCMS-8060 CL triple-quadrupole mass spectrometer (Simkon, Budapest, Hungary) was employed. Instrument control and data acquisition were performed using the LabSolutions CL (version 1.1) software. Chromatographic separation was accomplished using a Phenomenex Kinetex XB-C18, 50 × 2.1 mm (particle size 1.7 µm) stationary phase (Gen-Lab, Budapest, Hungary). The column temperature was set to 40 °C. The mobile phases were LC-MS grade water-formic acid 99.9:0.1 (*v*/*v*, mobile phase A) and methanol–formic acid 99.9:0.1 (*v*/*v*, mobile phase B). The following gradient program was applied (% mobile phase B): initial, 30%; 0.50 min, 30%; 3.00 min, 50%; 3.01 min, 90%; 5.50 min, 90%; and 5.51 min, 30%. The mobile phase flow rate was 0.25 mL/min, and the injected sample volume was 1.0 µL. The total run time was 7.00 min.

Mass spectrometry was performed using positive electrospray ionization and multiple reaction monitoring. Following the selection of the precursor [M+H]^+^ and of the product ions, the detection of mass transitions was optimized by the instrument control software by adjusting quadrupole 1 bias, the collision energy, and quadrupole 3 bias. The positive-mode multiple-reaction monitoring-optimization reports of the analytes are provided in Supplementary File S1. The ion transitions of the internal standards providing optimally sensitive signal intensities were found by conducting chromatographic runs under the conditions described above where the precursor ions were defined as those of the analytes plus five (i.e., *m*/*z* = 445.9 for ^2^H_5_-IBR and *m*/*z* = 479.7 for ^2^H_5_-DIB), and the signal intensities of the product ions were monitored in the mass range starting with the masses of the target product ions of the analytes (*m*/*z* = 304.1), and ending with *m*/*z* = 309.1. The optimized mass spectrometry settings are summarized in Supplementary File S1.

### 2.4. Quantitation

Plasma samples spiked with known concentrations of the analytes were employed for calibration. Each calibration set contained 6–8 concentration levels of IBR and DIB. The target values were 0.2, 1.0, 2.5, 10, 40, 80, 100, and 150 ng/mL, with two additional calibrator samples (320 and 520 ng/mL) run on a single occasion for evaluating plasma samples spiked with IBR and DIB at concentrations higher than 150 ng/mL. These values corresponded to 0.454, 2.27, 5.68, 22.7, 90.8, 182, 227, 341, 726, and 1180 nmol/L for IBR and 0.422, 2.11, 5.27, 21.1, 84.3, 169, 211, 317, 674, and 1096 nmol/L for DIB. Calibration was performed at the beginning of each batch run by spiking pooled blank plasma in which the absence of the analytes had been verified earlier. Calibration models were established using 1/concentration^2^-weighted linear regression.

The volumes of the analyte solutions spiked to calibrator and spiked plasma samples did not exceed 5% of that of plasma. Each calibrator, spiked plasma, and patient sample was measured in a single repeat.

### 2.5. Method Validation

Human plasma, separated from whole blood collected into 3-mL phlebotomy tubes containing tripotassium ethylene diamine tetraacetate (K_3_-EDTA) as anticoagulant and left over from routine laboratory diagnostic tests, was provided by the Central Laboratory, Department of Laboratory Medicine, Semmelweis University following irreversible deidentification. A total of 110 deidentified plasma samples were used, 10 of which had been pooled for preparing the calibrators and for performing selectivity and sample carryover tests. No interaction was made with the donors of these samples. All deidentified samples underwent analysis before spiking to confirm the absence of IBR and DIB.

The developed method was validated by evaluating selectivity, sample carryover, the performance of calibration models, assay accuracy and imprecision (by establishing assay error equations), matrix effect, and the stability of IBR and DIB in whole blood and plasma [26].

Selectivity and sample carryover were evaluated by comparing the chromatographic peak areas obtained with the highest-level calibrators to those recorded in blank plasma, injected alternately in three cycles. The performance of calibration curves was assessed by back-calculating the accuracies of measured calibrator concentrations. No lower limits of quantitation (LLOQ) were defined, as one of the objectives of constructing assay error equations is to provide quantitative estimates of the SD all the way down to zero analyte concentration. This strategy allows the laboratory to report all TDM results in a pharmacokinetically informative manner, and without censoring sub-LLOQ assay results that may otherwise be important clinically [18,19].

Assay error equations were established by spiking a total of 100 independent plasma samples with the analytes in five experiments performed on separate days. Four spiking levels were prepared in each experiment, adding up to a total of 20 spiking levels in addition to the blanks. Twenty independent plasma samples were spiked at each concentration level. Equations were defined for IBR, DIB, and the active moiety.

Matrix factors corrected with the peak areas of the internal standards (IMF) were determined at two concentration levels (2.0 and 80 ng/mL for each analyte, i.e., 4.54 nmol/L and 182 nmol/L for IBR and 4.21 nmol/L and 169 nmol/L for DIB, respectively). To this end, 5.0 µL of the analyte solutions (12 ng/mL or 480 ng/mL) and of a 336 ng/mL IS solution, prepared in methanol, were added to 140 µL supernatant obtained following the deproteinization of 50 µL blank plasma using 200 µL acetonitrile as described in Section 2.2. Six independent plasma samples were processed. The reference solutions were 140 µL acetonitrile-water 4:1 (*v*/*v*) mixtures spiked as described above. The mixtures and the reference solutions were subsequently diluted with 90 µL water. Internal standard-corrected matrix factors were calculated as the peak area ratios of the analytes and the internal standards in prepared plasma versus those in a neat solution. In the stability studies, the recoveries of IBR and DIB were calculated as the ratio of the concentrations measured after incubation and those measured at the beginning of the study. The analytes were considered stable at time points where recoveries exceeded 85.0%.

The preanalytical stability of IBR and DIB was evaluated by adding 20 µL methanol solutions containing 500 ng/mL IBR and DIB to two 1.0 mL aliquots of blood freshly drawn into phlebotomy tubes containing K_3_-EDTA. Three samples, 3 mL each, were taken from three healthy volunteers (manuscript authors G.B.K., I.V. and Z.K.). One of the fractions was centrifuged at 3000 rpm and 10 °C for 10 min immediately after spiking the analytes, and plasma was separated. Both spiked whole blood and the separated plasma were kept at ambient temperature for 6 h. At 0, 30, 60, 90, 180, and 360 min after sampling, whole blood was gently rotated five times and 150 µL whole blood pipetted into a 1.5 mL microcentrifuge tube that was subsequently centrifuged at 3000 rpm and 10 °C for 10 min. Fifty microliters was drawn from the supernatant of the whole blood sample as well as from the plasma, and was processed as described in Section 2.2.

### 2.6. Proof-of-Concept Experiments

In order to provide a proof of concept, IBR and DIB were assayed in the plasma samples of three patients treated with IBR. This evaluation was undertaken as part of a larger clinical study (ethical approval: 45371-2/2016/EKU, issued by the Scientific and Research Ethics Committee of the National Medical Research Council, Budapest, Hungary, Supplementary File S2). The criteria for inclusion were (1) age of ≥18 years, (2) treatment ongoing with IBR for more than 10 days, and (3) no concurrent administration of medications undergoing CYP3A4 metabolism. Detailed demographic and clinical information concerning the three participants is provided in Table 1. The subjects gave their written informed consent. Each participant took either two or three 140 mg Imbruvica capsules, as per the therapeutic provision, in the presence of the recruiting clinician. Blood was collected from the antecubital vein in a standard phlebotomy process by trained personnel into 3 mL tubes containing K_3_-EDTA at 0.5 h, 1 h, 2 h, 4 h, 23 h and 24 h postdose.

Blood samples were centrifuged at 3000 rpm for 10 min in a Hettich Universal 320R centrifuge (Auro-Science, Budapest, Hungary) at 10 °C. Plasma was separated and frozen at −70 °C until analysis was done within 2 weeks.

### 2.7. Data Evaluation

Data management and basic calculations were performed using Microsoft Excel 2016. Statistical evaluation was conducted in the R environment (version 4.0.5, 31 March 2021) using the following packages: “stats”, “AICcmodavg”, “NonCompart”, and “ncar”. Plots were created using the free academic version of ACD/ChemSketch (ACD Labs, Toronto, ON, Canada), Microsoft Excel, and the “ggplot2” package of R [27].

Assay accuracy was calculated as the ratio of the mean observed analyte concentration and the nominal concentration. Assay error equations were generated using four algorithms: Theil’s regression with and without the Siegel estimator, as well as unweighted linear or second-degree polynomial least squares regression. A script written by one of the authors (G.B.K.) in the R environment was employed to perform the calculations (Supplementary File S3) [21]. The goodness of the fitted assay error equations was quantified as the normalized sums of the squared residuals (NSSR) using the following formula:

(1) $$NSSR=\sum_{i=1}^{m}\frac{{\left(SD_{observed,i}-SD_{predicted,i}\right)}^{2}}{SD_{predicted,i}{}^{2}}$$

where *SD_observed,i_* is the observed *SD *of the concentrations measured in spiked plasma samples containing the analytes at the *i*-th spiking level, *SD_predicted,i_* is the estimate of the *SD* of the analyte concentration at the *i*-th spiking level, as inferred from the fitted regression equation, and *m* is the number of spiking levels (*m* = 20) [21].

Noncompartmental pharmacokinetic calculations were performed for IBR and for the active moiety (IBR + DIB) based on the concentration series obtained in the three participants receiving IBR. The AUC() function of the “NonCompart” as well as the pdfNCA() function of the “ncar” package were used in the R environment with default settings. Since the subjects were in steady state concerning IBR and DIB concentration profiles, the 24 h concentrations were also employed for simulating 0 h predose levels. The employed R packages “NonCompart” and “ncar” are compatible with the Study Data Tabulation Model-formatted dataset of the Clinical Data Interchange Standards Consortium standard, and their performance had previously been demonstrated to yield results equivalent to those obtained using leading commercial pharmacokinetic modeling software [28,29].

## 3. Results

### 3.1. Bioanalytical Method Validation

IBR and DIB were eluted from the stationary phase as symmetrical peaks with retention times of 4.3 min and 4.2 min, respectively. The internal standards were eluted with the same retention times as their unlabeled analogues. The method was sensitive and selective, with no sample carryover observed (Supplementary File S4). The relationship between the concentrations of IBR and DIB and the analyte/internal standard peak area ratios was linear in the calibrated concentration range.

Method accuracy and precision are presented in Table 2. In the calibrated concentration range, the accuracy was 99.4–110% and 91.7–118%, while the relative standard deviation was 1.88–6.04% and 0.59–27.3% for IBR and DIB, respectively. Bioanalytical method-validation guideline criteria (accuracy 85–115%, or 80–120% at the lower limit of quantitation, relative standard deviation <15%, or <20% at the lower limit of quantitation) were met for IBR in the entire calibrated range, and for DIB at 11.3–1096 nmol/L [26]. The accuracy of the assay was 120–139% and 92.1–339%, with relative SDs of 8.3–16.3% and 11.9–54.2%, respectively, under the calibrated concentration range. The internal standard-corrected matrix factors were 92.3% (10.4%) and 103% (6.4%) for IBR, and 115% (12.4%) and 101% (6.0%) for DIB (Table 3).

### 3.2. Stability of IBR and DIB in the Early Preanalytical Phase

**Figure 2:**
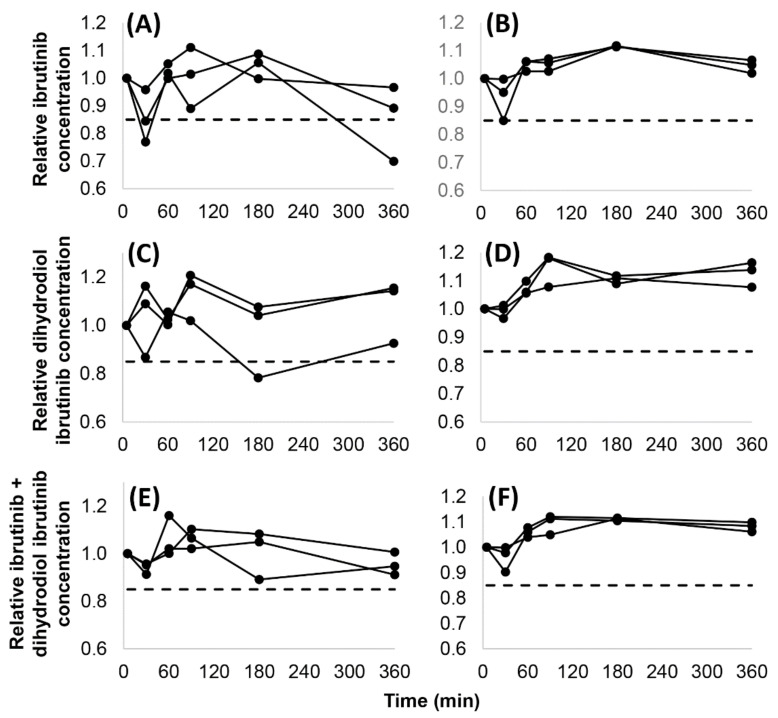
Stability of ibrutinib (**A**,**B**), dihydrodiol ibrutinib (**C**,**D**) and the active moiety (sum of ibrutinib and dihydrodiol ibrutinib concentrations) (**E**,**F**) in whole blood (**A**,**C**,**E**) and in plasma (**B**,**D**,**F**) at 25 °C over 6 h in 3 independent samples. The dashed line (**- - -**) displays the limit for judging analyte stability as acceptable (0.85).

Fifteen analyses of IBR and DIB (3 observations at 5 time points) were conducted in whole blood and in plasma. In whole blood, recoveries lower than 85% were obtained in two cases and in one case concerning IBR and DIB, respectively, with only one of these occurring after 6 h, and with no identifiable trends of the recoveries seen. The recovery of the active moiety (IBR + DIB) exceeded 85% in all cases. In plasma, 85% recoveries or higher were attained in all analyses. The dispersion of the measured concentrations was larger in whole blood than in plasma, indicating that binding to cell components may have influenced the analytical results. The recoveries (t = 0 min: 100%) are shown in Figure 2.

### 3.3. Assay Error Equations of IBR, DIB, and the Active Moiety (IBR + DIB)

**Figure 3:**
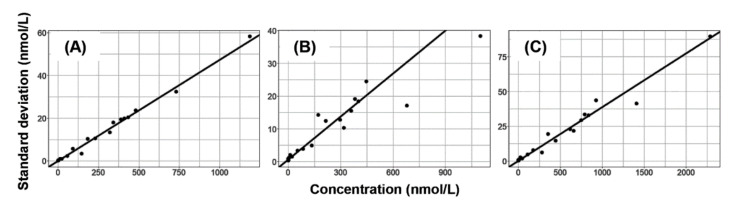
Linear regression applied to the concentration–standard deviation relationships using Theil’s regression with the Siegel estimator. (**A**) Ibrutinib (standard deviation = 0.04721 × concentration + 0.05559. (**B**) Dihydrodiol ibrutinib (standard deviation = 0.04382 × concentration + 0.6814). (**C**) Active moiety (standard deviation = 0.03854 × sum of IBR + DIB concentrations + 0.3526).

The results of various types of regression performed on the concentration–SD relationships are summarized in Table 4. The performance of unweighted linear least squares was unacceptable for IBR, as the predicted SDs were lower than 0 up to 5.96 nmol/L, with a negative intercept (−0.1285). The nonlinear coefficients of the unweighted second-degree least squares polynomials were <0.0001, confirming the linearity of the relationships. Theil’s regression with the Siegel estimator delivered the best overall performance in view of the consistently low NSSR values, and of the low yet positive intercepts obtained. The assay error equations obtained using this algorithm were SD = 0.04721 × concentration + 0.05559 (IBR), SD = 0.04382 × concentration + 0.6814 (DIB) and SD = 0.03854 × sum of IBR + DIB concentrations + 0.3526 (active moiety, Figure 3). In the case of DIB and the active moiety, the differences in the performance of the four regression approaches were negligible.

### 3.4. 24 h Therapeutic Monitoring of IBR and DIB in the Plasma of Chronic Lymphocyte Leukemia Patients Receiving IBR

**Figure 4:**
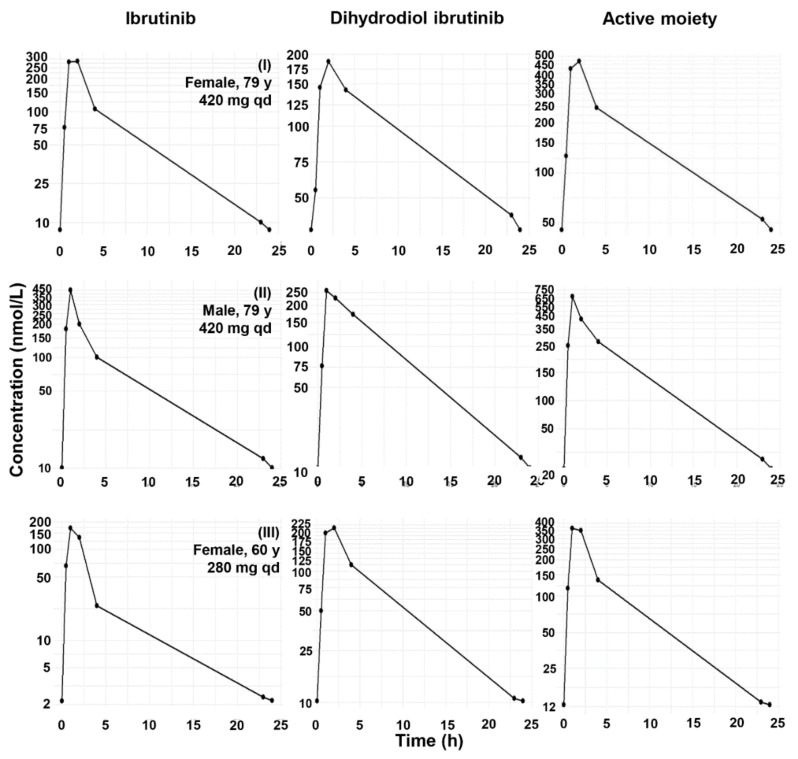
24 h steady-state concentration profiles of IBR, DIB, and the active moiety (IBR + DIB) in three chronic lymphocyte leukemia patients taking 420 mg (Subjects **I** and **II**) or 280 mg (Subject **III**) IBR per day.

The concentration profiles of IBR and DIB obtained in adult chronic lymphocyte leukemia patients are displayed in Figure 4. Maximum concentrations of IBR, and also of DIB, were attained not later than 2 h after drug intake. The mean DIB/IBR concentration ratios were 0.96–1.19 (SD: 0.39–0.60) between 0.5–2 h, 2.36 ± 1.69 at 4 h, and 3.34–3.44 (SD: 1.47–1.56) at the trough (23–24 h). The primary determinant of the maximum concentrations and the areas under the concentration–time curves (AUC) was the dose. Noncompartmental pharmacokinetic characteristics calculated from these curves are shown in Table 5 (the reports of the evaluations are provided in Supplementary File S5).

## 4. Discussion

Few publications have discussed the bioanalysis and the pharmacokinetics of IBR, especially together with its major active metabolite DIB, in humans. The available methodologies have been reviewed extensively [30]. So far, LC-MS/MS has been the only technology to be used for the simultaneous analysis of IBR and DIB, with positive electrospray ionization and the selection of the pseudomolecular ions as precursors. Taurocholic acid, which appears in the bloodstream at higher concentrations in patients with hepatic impairment, has been identified to interfere, requiring its separation from DIB either chromatographically or by high-resolution mass spectrometry [31]. Deproteinization with acetonitrile and solvent exchange have been selected most often for preparing samples [8,25,32,33,34]. Others have employed simplified liquid extraction and solvent exchange [31,35]. In a single case, liquid–liquid extraction was performed [36]. An approach to the simple and rapid analysis of IBR and DIB in cerebrospinal fluid has also been published [33]. The starting sample volume was 20–200 µL in all of these works.

The presented bioanalytical method, which has been implemented successfully for routine TDM in our laboratory, has been designed specifically to support clinical pharmacokinetic modeling and precision pharmacotherapy. The method allows the rapid assessment of IBR and DIB concentrations as components of a broader panel of tyrosine kinase inhibitors. The employed high-throughput approach relies on sample preparation consisting of two rapid, cost-efficient steps: deproteinization with acetonitrile and dilution. The preparation of a full 96-well plate for analysis requires less than 1 h. With an analysis time of 7 min, up to 170 test results can be reported within 24 h. Provided the 3-month stability of IBR and DIB in plasma at −70 °C and the lack of availability of CE-IVD lyophilized plasma controls, blank plasma spiked at various concentrations or patient samples collected before drug intake and at 1.5 h postdose can serve as control samples for the analysis. In our routine assays, we used plasma spiked at 10 ng/mL and 100 ng/mL to this end. As the range of drugs with a clinical demand to monitor their concentrations in patients belonging to high-risk populations is growing rapidly, the use of in-house calibrators and internal controls is becoming more common and accepted in the absence of commercially available preparations [37].

Recently, the differential absorption of IBR from its isotopically labeled analogues to polymeric surfaces, including the walls of containers used during sample preparation and the polyether ether ketone components of LC-MS/MS systems, has been reported by Mzik et al. [38]. This differential absorption of the analyte led to large SDs and remarkable carryover at low concentrations (0.25 ng/mL, corresponding to 0.567 nmol/L). The SDs of IBR obtained in our study did not confirm this finding, with the relative SDs never exceeding 16.3% from as low as 0.488 nmol/L. In addition, we did not observe appreciable carryover of IBR or DIB in our experiments. Nevertheless, Mzik et al. demonstrated that the components of the employed liquid chromatograph may have a profound impact on this phenomenon. Therefore, a potential reason for this discrepancy is that inside the liquid chromatograph used in our research, the analytes could only get in contact with plastic material after being eluted from the chromatographic column by an eluent composition containing a relatively high fraction of organic component. In addition, we used methanol as the organic solvent, while Mzik et al. used acetonitrile, a less potent solvent for IBR.

It should be noted that the RSDs we recorded for DIB exceeded 20% at all spiking levels, except one in the range of 0.453–5.56 nmol/L (0.217–2.67 ng/mL). In addition, RSDs changed stepwise from 45.8–54.2% (0.453 and 0.906 nmol/L) to 11.9–23.5% (1.89–11.3 nmol/L) and then to 0.59–8.78% (all spiking levels higher than 11.3 nmol/L). While the octanol–water partitioning of DIB is similar to that of IBR, the presence of a primary and a secondary hydroxyl group in the structure may increase the affinity of DIB to slightly polar polymeric surfaces. In conclusion, the sharp differences between the RSDs obtained for IBR and DIB and the fact that large RSDs were obtained for DIB only at low concentrations support the assumption of underlying causes similar to those described by Mzik et al.

The stability of IBR and DIB in whole blood and plasma kept at ambient temperature for 6 h was acceptable for clinical use. The recoveries of DIB were higher than those of IBR at late time points, probably as a result of IBR’s covalent binding to endogenous thiols, such as glutathione [39]. Huynh et al. found that the degradation of IBR was considerable (with recoveries of 46.7–72.9%) after plasma was kept at ambient temperature for 24 h [24]. Recoveries of 87–100% were reported after keeping plasma at 0 °C for 2 h or at −80 °C for 2 months, as well as following multiple freeze–thaw cycles. Thermostatting the autosampler tray at 4 °C for 48 h resulted in all reanalyses yielding results within the 85–115% relative concentration range (80–120% at LLOQ), the recommended range of acceptability according to international bioanalytical method-validation guidelines [26]. It can be concluded that blood samples collected for the analysis of IBR and DIB should be centrifuged as soon as possible, preferably within 6 h, and the supernatant should be separated and kept frozen until the analysis.

The assay error equation is the experimental basis for determining the optimal weight (1/variance, also called the Fisher information) of each observation employed for nonlinear curve fitting during the construction of the pharmacokinetic model. Based on mathematical theory and the obtained unbiased NSSR indicators, Theil’s regression with the Siegel estimator, a nonparametric linear regression method, which is 100% resistant to outliers regarding the identification of a linear trend, was the most consistently accurate for describing the quantitative relationship between IBR and DIB concentrations and assay SD. A disadvantage of the unweighted (ordinary) least squares method, also demonstrated by our results, is that negative SDs, which are nonsense, are frequently predicted for concentrations below the lower limit of the calibrated concentration range. Theil’s regression with the Siegel estimator, on the other hand, is entirely resistant to the outliers of any linear trend and does not yield negative intercepts when evaluating concentration–SD relationships obtained by applying methods based on LC-MS/MS and the use of internal standards.

While determining the assay error equation experimentally is closely linked to the construction of nonparametric pharmacokinetic models, it also brings other important benefits. Assay accuracy and precision is evaluated by running 400 samples (in addition to the blanks) from zero concentration to the high end of the calibrated concentration range, in contrast to assaying 24 samples typically in a conventional within-run (intra-day) study and the reanalysis of a fraction of these in the between-run (interday) experiments. Concentration points can be retested and further concentration points can be added flexibly, providing a suitable context for partial method revalidation with an experimentally established SD acceptability range. In addition, knowledge of the SD and accepting that it can be relatively high at low concentrations allows the laboratory to avoid the use of a lower numerical limit for reporting drug and metabolite levels.

The pharmacokinetic values obtained in the three CLL patients on IBR were overall comparable to earlier findings [10,31,32]. It is also apparent that, due to the high concentrations it attains, the inclusion of DIB in the models, either as a metabolite or a component of the active moiety, is crucial.

Individualized pharmacotherapy relying on model-informed precision dosing is a multidisciplinary approach. In order to provide reliable and justified reports for supporting individual decisions, TDM laboratories need to exert dedicated knowledge and activities (an exception is when precision dosing is based on the use of physiologically based pharmacokinetic–pharmacodynamic models that do not require continuous TDM [40]). Failure to establish and periodically revise an experimentally determined error model or to be informed on the stability of the analytes is likely to contribute to the enormous differences in the pharmacokinetic estimates made by various research groups. Nevertheless, the maintenance of such dedicated TDM laboratories is affordable mainly to academic facilities, presenting a very large barrier to the broader application of model-informed precision dosing. It must also be emphasized that model-informed precision dosing is not equivalent to TDM or to population pharmacokinetic modeling.

A limitation of this research is that the direct application of the developed methodology to constructing nonparametric pharmacokinetic models of IBR, DIB, and the active moiety (IBR + DIB) or the clinical validation of these models could not be accomplished, due to the small number of available patients. In addition, evaluating the method using other types of blood samples should be useful for the optimization of method performance.

## 5. Conclusions

The presented high-throughput methodology allows TDM laboratories to assist precision pharmacotherapy with IBR efficiently. Blood samples are recommended to be centrifuged, with the supernatant separated and cooled no later than 6 h following sample collection. DIB concentrations should be monitored along with the parent drug. The presented assay error equations can be employed for estimating the imprecision of nonparametric pharmacokinetic models of IBR, DIB, and the active moiety (IBR + DIB). The correct timing of sample collection related to dosing is essential to capture information relevant for constructing efficient pharmacokinetic models of IBR, DIB, and IBR + DIB.

## Figures and Tables

**Table 1:** Demographic and clinical characteristics of the patients receiving IBR. CLL, chronic lymphocytic leukemia.

	Participant 1	Participant 2	Participant 3
Gender	female	male	female
Age	79	79	60
Diagnosis	CLL	CLL	CLL
Reported co-morbidities	melanoma malignum, hypertension	none	resected gall-bladder
Ibrutinib daily dose	420 mg	420 mg	280 mg
eGFR (mL/min/1.73 m^2^)	50.9	57.6	>90
glutaryl oxaloacetate transaminase (U/L)	18	20	17
glutaryl pyruvate transaminase (U/L)	12	13	14
gamma-glutamyl transferase (U/L)	23	12	20
white blood cell count (G/L)	2.7	27.8	241
neutrophile (%)	59.2	0.0	0.0
eosinophile (%)	0.4	0.0	0.0
basophile (%)	1.5	0.0	0.0
monocyte (%)	9.6	0.0	0.0
lymphocyte (%)	29.3	0.0	0.0
immature granulocyte (%)	9.3	0.0	0.1

**Table 2:** Performance of the assay method. The analytes were spiked to 20 independent human plasma samples at each concentration level. Experiments were conducted on five different days, indicated by different colors. N/D, not determined. RSD, relative standard deviation. SD, standard deviation. Experiments performed on different days are shown in different colors.

Ibrutinib				Dihydrodiol Ibrutinib			
Concentration (nmol/L)	Accuracy (%)	SD (nmol/L)	RSD	Concentration (nmol/L)	Accuracy (%)	SD (nmol/L)	RSD
0.488	139	0.068	10.0%	0.453	339	0.830	54.2%
0.976	134	0.213	16.3%	0.906	209	0.864	45.8%
1.99	120	0.200	8.31%	1.85	137	0.299	11.9%
2.30	101	0.091	3.91%	2.15	95.4	0.561	27.3%
5.96	102	0.325	5.33%	5.56	92.1	1.20	23.5%
12.2	104	0.334	2.63%	11.3	118	1.98	18.1%
23.0	99.3	0.904	3.96%	21.5	94.1	1.43	7.06%
57.2	99.2	3.43	6.04%	52.9	114	3.29	0.59%
92.0	104	5.59	5.85%	85.9	92.8	3.88	4.86%
146	106	0.386	1.88%	136	116	4.85	1.20%
184	107	10.3	5.19%	172	94.2	14.2	8.78%
230	108	10.6	4.24%	215	91.7	12.4	6.31%
320	108	13.3	3.84%	297	108	12.7	3.96%
343	107	17.9	4.88%	318	110	10.3	2.94%
388	110	19.4	4.55%	360	112	15.4	3.82%
411	106	19.7	4.52%	381	110	19.1	4.53%
434	107	20.3	4.39%	403	112	18.4	4.06%
481	102	23.6	4.80%	447	115	24.4	4.76%
731	99.4	32.3	4.45%	649	105	17.1	2.40%
1187	106	58.2	4.62%	1100	107	38.4	3.25%

**Table 3:** Internal standard-corrected matrix factors of ibrutinib and dihydrodiol ibrutinib. Six independent human serum matrices (A-F) and two spiking levels were used. SD, standard deviation. RSD, relative standard deviation.

Matrix Identifier	Ibrutinib		Dihydrodiol Ibrutinib	
	Low Level: 2.0 ng/mL (4.54 nmol/L)	High Level: 80 ng/mL (182 nmol/L)	Low Level: 2.0 ng/mL (4.21 nmol/L)	High Level: 80 ng/mL (169 nmol/L)
**A**	0.894	1.002	1.226	0.942
**B**	1.069	0.975	0.920	0.993
**C**	0.906	1.073	1.039	1.044
**D**	0.815	1.014	1.278	1.009
**E**	0.849	1.131	1.267	1.104
**F**	1.004	0.958	1.173	0.952
**Mean ± SD**	0.923 ± 0.096	1.03 ± 0.065	1.15 ± 0.143	1.01 ± 6.0
**RSD (%)**	10.4	6.4	12.4	6.4

**Table 4:** Performance of regression algorithms applied to the concentration–standard deviation relationships. IBR, ibrutinib. DIB, dihydrodiol ibrutinib. IBR + DIB, sum of IBR and DIB concentrations (active moiety). NSSR, normalized sum of squared residuals. OLS, unweighted linear least squares. 2nd LS, unweighted 2nd-degree least squares. Siegel, Theil’s regression with the Siegel estimator. Theil, Theil’s regression.

Algorithm	NSSR			Slope			Intercept		
	IBR	DIB	IBR + DIB	IBR	DIB	IBR + DIB	IBR	DIB	IBR + DIB
Theil	1.876	3.567	3.386	0.0479	0.0418	0.0387	0.06635	0.5308	0.4115
Siegel	2.352	2.516	4.682	0.0472	0.0438	0.0385	0.05559	0.6814	0.3526
OLS	106.9	4.428	1.986	0.0480	0.0342	0.0373	−0.1285	1.970	0.6084
2nd LS	1.667	2.615	1.934	0.0457 *	0.0447 *	0.0359 *	0.08408	1.071	0.8606

* Linear coefficients are shown.

**Table 5:** Calculated individual pharmacokinetic properties of IBR and the active moiety (IBR + DIB). AUC_0–24_, area under the concentration–time curve from dose intake to 24 h postdose. AUMC_0–24_, area under the first moment of the concentration–time curve from dose intake to 24 h postdose. CL/F, apparent clearance. c_max_, peak concentration. K_e_, terminal elimination rate constant. MRT_0–24_, mean residence time from dose intake to 24 h postdose. t_1/2_, systemic half-life. t_max_, time to reach the peak concentration. V/F, apparent volume of distribution.

Parameter	Ibrutinib			Dihydrodiol Ibrutinib			Ibrutinib + Dihydrodiol Ibrutinib		
	Patient 1	Patient 2	Patient 3	Patient 1	Patient 2	Patient 3	Patient 1	Patient 2	Patient 3
AUC_0–24_ (nmol × L/h)	1786	1740	613	2347	2528	1800	4134	4268	2414
AUMC_0–24_ (nmol × L)	7488	7434	2051	16,593	11,626	8172	24,082	19,071	10,230
c_max_ (nmol/L)	265.6	374.0	163.2	184.7	253.7	216.6	450.3	627.7	358.3
Dose-normalized c_max_ [nmol/(L × mmol)]	278.7	392.4	256.6	Cannot be calculated			472.5	658.6	563.3
t_max_ (h)	2.0	1.0	1.0	2.0	1.0	2.0	2.0	1.0	1.0
CL/F (L/h)	515	523	1008	Cannot be calculated			Cannot be calculated		
MRT_0–24_ (h)	4.19	4.28	3.34	7.07	4.60	4.54	5.82	4.47	4.24
k_e_ (1/h)	0.126	0.113	0.121	0.069	0.130	0.122	0.085	0.123	0.122
t_1/2_ (h)	5.49	6.12	5.74	10.1	5.35	5.68	8.13	5.64	5.70
V/F (L)	4080	4620	8346	Cannot be calculated			Cannot be calculated		

## Data Availability

Additional data are available from the corresponding author on reasonable request.

## Supplementary Materials

The following supporting information can be downloaded at: https://www.mdpi.com/article/10.3390/molecules27154766/s1, Supplementary File S1: Multiple reaction monitoring optimization reports and the final detection settings employed for the high-throughput clinical analysis of ibrutinib and dihydrodiol ibrutinib. Note that the plus (‘+’) signs in the “Optimality condition” sections refer to employing positive polarity. Under this setting, the instrument control software displays negative values for Q1 Pre-bias, CE and Q3 Pre-bias. Supplementary File S2: Electronic copy of the ethical approval 45371-2/2016/EKU. Supplementary File S3: Scripts run in the R environment. Supplementary File S4: Representative ion chromatograms of ibrutinib (IBR), dihydrodiol ibrutinib (DIB), and their respective internal standards ^2^H_5_-ibrutinib (^2^H_5_-IBR) and ^2^H_5_-dihydrodiol ibrutinib (^2^H_5_-DIB). (A), IBR in the level 1 calibration sample (2.27 nmol/L), (B) ^2^H_5_-IBR in the level 1 calibration sample, (C) IBR in a blank sample, (D) ^2^H_5_-IBR in a blank sample, (E) DIB in the level 1 calibration sample (2.11 nmol/L), (F) ^2^H_5_-DIB in the level 1 calibration sample, (G) DIB in a blank sample, (H) ^2^H_5_-DIB in a blank sample. Supplementary File S5: Individual noncompartmental analysis results.

## Sample Availability

IBR and DIBR were purchased from a commercial supplier. Samples of the compounds are not available from the authors.
