# Supplementary material for: A High-Throughput Clinical Laboratory Methodology for the Therapeutic Monitoring of Ibrutinib and Dihydrodiol Ibrutinib
Source: Molecules. 2022 Jul 25;27(15):4766. doi: 10.3390/molecules27154766 (PMC9331678; doi:10.3390/molecules27154766)
Supplement: Supplementary file 1 [file molecules-27-04766-s001.zip › Supplementary File S1.pdf]

Supplementary File S1: Multiple reaction monitoring optimization reports and the final detection settings employed for the high-throughput clinical analysis of ibrutinib and dihydrodiol ibrutinib. Note that the plus ('+') signs in the "Optimality condition" sections refer to employing positive polarity. Under this setting, the instrument control software displays negative values for Q1 Pre-bias, CE and Q3 Pre-bias.

A. Optimization report of ibrutinib

Original Method File :C:\LabSolutions\Data\methods\optimization\clnnclzibr_step3_MRM-1.lcm

Tuning File :

<<Optimality condition>>

Adjust Precursor m/z :On

Optimization for Q1 Pre-rod Bias :On

Optimization for CE :On

Optimization for Q3 Pre-rod Bias :On

Adjust Product m/z :On

Optimization for CE in detail :On

ID Acq Mode +/- Sample ID Vial# Tray Inj Vol.

6 MRM + 3 1 0.5

7 MRM + 3 1 0.5

<<Summary of Optimum Result>>

#6 OK

#7 OK

<<Optimum result>>

ID6 Ch1

441.40>304.00 -> 440.90>304.10

Q1 Pre Bias -15.0 -> -10.0

CE -25.0 -> -31.0

Q3 Pre Bias -15.0 -> -15.0

ID7 Ch1

441.40>138.00 -> 440.90>138.10

Q1 Pre Bias -15.0 -> -10.0

CE -25.0 -> -28.0

Q3 Pre Bias -15.0 -> -14.0

<<Intensity>>

#6:(ibrutinib-304) Ch:1 Precursor

440.90	10074288	100.0%	Selected
441.00	8845818	87.8%	
441.10	6892959	68.4%	
441.20	4655507	46.2%	
441.30	1478205	14.7%	
441.40	753431	7.5%	
441.50	2152810	21.4%	
441.60	2041495	20.3%	
441.70	2170024	21.5%	
441.80	2633368	26.1%	
441.90	2907099	28.9%	

#6:(ibrutinib-304) Ch:1 CE

-50.00	172040	14.5%	
-45.00	391853	33.0%	
-40.00	735188	62.0%	
-35.00	1065577	89.8%	
-30.00	1185993	100.0%	Selected

-25.00	917267	77.3%	
-20.00	408958	34.5%	
-15.00	101741	8.6%	
-10.00	26750 2.3%		

#6:(ibrutinib-304) Ch:1 CE_Detail

-35.00	1080546	88.2%	
-34.00	1118544	91.4%	
-33.00	1166421	95.3%	
-32.00	1199020	97.9%	
-31.00	1224438	100.0%	Selected
-30.00	1195843	97.7%	
-29.00	1178201	96.2%	
-28.00	1146448	93.6%	
-27.00	1122142	91.6%	
-26.00	1022714	83.5%	
-25.00	939981	76.8%	

#6:(ibrutinib-304) Ch:1 Product

303.50	248066	20.6%	
303.60	360106	30.0%	
303.70	711172	59.2%	
303.80	1013840	84.4%	
303.90	1133140	94.3%	
304.00	1191496	99.2%	
304.10	1201696	100.0%	Selected
304.20	1087138	90.5%	
304.30	876628	72.9%	
304.40	373695	31.1%	

304.50	35354	2.9%

#6:(ibrutinib-304) Ch:1 Q1PreBias

-30.00	1197497	88.3%	
-29.00	1215264	89.6%	
-28.00	1247682	92.0%	
-27.00	1232652	90.9%	
-26.00	1165875	86.0%	
-25.00	1113461	82.1%	
-24.00	1077017	79.4%	
-23.00	1069989	78.9%	
-22.00	1117579	82.4%	
-21.00	1215368	89.6%	
-20.00	1261780	93.1%	
-19.00	1269784	93.7%	
-18.00	1206827	89.0%	
-17.00	1255946	92.6%	
-16.00	1337542	98.7%	
-15.00	1341513	99.0%	
-14.00	1210001	89.3%	
-13.00	1214209	89.6%	
-12.00	1338251	98.7%	
-11.00	1319949	97.4%	
-10.00	1355697	100.0%	Selected

#6:(ibrutinib-304) Ch:1 Q3PreBias

-30.00	1424839	90.2%
-29.00	1371043	86.8%
-28.00	1340724	84.8%

-27.00	1317466	83.4%	
-26.00	1305899	82.6%	
-25.00	1339712	84.8%	
-24.00	1378780	87.3%	
-23.00	1443065	91.3%	
-22.00	1494015	94.5%	
-21.00	1488250	94.2%	
-20.00	1400794	88.6%	
-19.00	1245006	78.8%	
-18.00	1145013	72.5%	
-17.00	1176401	74.4%	
-16.00	1438987	91.1%	
-15.00	1580227	100.0%	Selected
-14.00	1267684	80.2%	
-13.00	1138626	72.1%	
-12.00	1390226	88.0%	
-11.00	1233866	78.1%	
-10.00	1108890	70.2%	

#7:(ibrutinib-138) Ch:1 Precursor

440.90	10232870	100.0%	Selected
441.00	8996696	87.9%	
441.10	6964747	68.1%	
441.20	4674962	45.7%	
441.30	1505039	14.7%	
441.40	794368	7.8%	
441.50	2191437	21.4%	
441.60	2119591	20.7%	
441.70	2311052	22.6%	
441.80	2727065	26.7%	

441.90	2970545	29.0%	

#7:(ibrutinib-138)	Ch:1 CE		
-50.00	146395	9.6%	
-45.00	295647	19.3%	
-40.00	556324	36.4%	
-35.00	985323	64.4%	
-30.00	1505763	98.5%	
-25.00	1529157	100.0%	Selected
-20.00	808384	52.9%	
-15.00	191442	12.5%	
-10.00	37901 2.5%		

#7:(ibrutinib-138)	Ch:1 CE_Detail		
-30.00	1517026	92.8%	
-29.00	1587566	97.1%	
-28.00	1635084	100.0%	Selected
-27.00	1633383	99.9%	
-26.00	1615600	98.8%	
-25.00	1545797	94.5%	
-24.00	1415419	86.6%	
-23.00	1279101	78.2%	
-22.00	1126365	68.9%	
-21.00	950323	58.1%	
-20.00	819711	50.1%	

#7:(ibrutinib-138)	Ch:1 Product	
137.50	146650	8.5%

137.60	415550	24.0%	
137.70	773060	44.7%	
137.80	1178193	68.1%	
137.90	1469164	84.9%	
138.00	1637187	94.6%	
138.10	1730439	100.0%	Selected
138.20	1708889	98.8%	
138.30	1671206	96.6%	
138.40	1327024	76.7%	
138.50	183531	10.6%	

#7:(ibrutinib-138) Ch:1 Q1PreBias

-30.00	1572547	83.0%
-29.00	1666712	87.9%
-28.00	1659740	87.6%
-27.00	1660813	87.6%
-26.00	1586793	83.7%
-25.00	1531345	80.8%
-24.00	1478124	78.0%
-23.00	1474761	77.8%
-22.00	1550336	81.8%
-21.00	1641816	86.6%
-20.00	1724989	91.0%
-19.00	1723711	91.0%
-18.00	1703743	89.9%
-17.00	1725926	91.1%
-16.00	1874952	98.9%
-15.00	1859859	98.1%
-14.00	1623051	85.6%
-13.00	1659108	87.5%

-12.00	1864032	98.4%	
-11.00	1811609	95.6%	
-10.00	1895149	100.0%	Selected

#7:(ibrutinib-138) Ch:1 Q3PreBias

-30.00	2435547	92.5%	
-29.00	2467934	93.7%	
-28.00	2507671	95.2%	
-27.00	2504155	95.1%	
-26.00	2487594	94.4%	
-25.00	2491556	94.6%	
-24.00	2530565	96.1%	
-23.00	2492622	94.6%	
-22.00	2429631	92.2%	
-21.00	2382156	90.4%	
-20.00	2260953	85.8%	
-19.00	2178490	82.7%	
-18.00	2136194	81.1%	
-17.00	2209438	83.9%	
-16.00	2438053	92.6%	
-15.00	2615910	99.3%	
-14.00	2634198	100.0%	Selected
-13.00	2312334	87.8%	
-12.00	2019659	76.7%	
-11.00	2064760	78.4%	
-10.00	2511007	95.3%	

B. <u>Dihydrodiol ibrutinib</u>

Original Method File :C:\LabSolutions\Data\methods\optimization\dibr_step3_MRM-190625-1.lcm

Tuning File :

<<Optimality condition>>

Adjust Precursor m/z :On

Optimization for Q1 Pre-rod Bias :On

Optimization for CE :On

Optimization for Q3 Pre-rod Bias :On

Adjust Product m/z :On

Optimization for CE in detail :On

ID	Acq Mode	+/-	Sample ID	Vial#	Tray	Inj Vol.
1	MRM	+	3	0	0.1	
2	MRM	+	3	0	0.1	

<<Summary of Optimum Result>>

#1 OK

#2 OK

<<Optimum result>>

ID1 Ch1

474.90>304.50 -> 474.70>304.10

Q1 Pre Bias -11.0 -> -13.0

CE -25.0 -> -32.0

Q3 Pre Bias -15.0 -> -15.0

ID2 Ch1

475.00>84.10 -> 474.70>84.10

Q1 Pre Bias -11.0 -> -16.0

CE -25.0 -> -39.0

Q3 Pre Bias -16.0 -> -15.0

<<Intensity>>

#1:(dibr-305) Ch:1 Precursor

474.40	921631	25.3%	
474.50	2493502	68.6%	
474.60	3381551	93.0%	
474.70	3635650	100.0%	Selected
474.80	3561682	98.0%	
474.90	3433977	94.5%	
475.00	3289134	90.5%	
475.10	2901561	79.8%	
475.20	1832063	50.4%	
475.30	940280	25.9%	
475.40	557080	15.3%	

#1:(dibr-305) Ch:1 CE

-50.00	2878	28.3%	
-45.00	5212	51.3%	
-40.00	8635	84.9%	
-35.00	10169	100.0%	Selected
-30.00	8524	83.8%	
-25.00	5345	52.6%	
-20.00	2300	22.6%	
-15.00	622	6.1%	
-10.00	133	1.3%	

#1:(dibr-305) Ch:1 CE_Detail

-40.00	8691	83.0%	
-39.00	7769	74.2%	
-38.00	8068	77.1%	
-37.00	9280	88.6%	
-36.00	9981	95.3%	
-35.00	9403	89.8%	
-34.00	10247	97.9%	
-33.00	10314	98.5%	
-32.00	10470	100.0%	Selected
-31.00	9202	87.9%	
-30.00	8702	83.1%	

#1:(dibr-305) Ch:1 Product

304.00	428862	97.7%	
304.10	439028	100.0%	Selected
304.20	385751	87.9%	
304.30	285760	65.1%	
304.40	82524	18.8%	
304.50	8846	2.0%	
304.60	2344	0.5%	
304.70	1822	0.4%	
304.80	1811	0.4%	
304.90	1967	0.4%	
305.00	1656	0.4%	

#1:(dibr-305) Ch:1 Q1PreBias

-30.00	426977	89.4%
-29.00	427262	89.4%
-28.00	429472	89.9%

-27.00	402278	84.2%	
-26.00	392064	82.1%	
-25.00	408258	85.5%	
-24.00	407509	85.3%	
-23.00	428510	89.7%	
-22.00	450336	94.3%	
-21.00	451318	94.5%	
-20.00	438394	91.8%	
-19.00	430449	90.1%	
-18.00	429284	89.9%	
-17.00	465585	97.5%	
-16.00	471779	98.8%	
-15.00	438243	91.7%	
-14.00	425502	89.1%	
-13.00	477724	100.0%	Selected
-12.00	459941	96.3%	
-11.00	467570	97.9%	
-10.00	443489	92.8%	

#1:(dibr-305) Ch:1 Q3PreBias

-30.00	492009	86.7%
-29.00	465454	82.0%
-28.00	462210	81.4%
-27.00	443233	78.1%
-26.00	455765	80.3%
-25.00	466670	82.2%
-24.00	499156	87.9%
-23.00	504199	88.8%
-22.00	514099	90.5%
-21.00	516008	90.9%

-20.00	488002	85.9%	
-19.00	437555	77.1%	
-18.00	374029	65.9%	
-17.00	411145	72.4%	
-16.00	507687	89.4%	
-15.00	567790	100.0%	Selected
-14.00	433857	76.4%	
-13.00	379701	66.9%	
-12.00	484569	85.3%	
-11.00	430619	75.8%	
-10.00	374673	66.0%	

#2:(dibr-84) Ch:1 Precursor

474.50	2568301	69.5%	
474.60	3405801	92.1%	
474.70	3697734	100.0%	Selected
474.80	3614518	97.7%	
474.90	3521096	95.2%	
475.00	3357427	90.8%	
475.10	2903618	78.5%	
475.20	1845298	49.9%	
475.30	953242	25.8%	
475.40	585441	15.8%	
475.50	922194	24.9%	

#2:(dibr-84) Ch:1 CE

-50.00	208093	87.9%	
-45.00	228575	96.5%	
-40.00	236783	100.0%	Selected

-35.00	221076	93.4%	
-30.00	166670	70.4%	
-25.00	72789	30.7%	
-20.00	15439	6.5%	
-15.00	2100	0.9%	
-10.00	767	0.3%	

#2:(dibr-84) Ch:1 CE_Detail

-45.00	234493	93.4%	
-44.00	241707	96.3%	
-43.00	235425	93.8%	
-42.00	245007	97.6%	
-41.00	243076	96.9%	
-40.00	240778	96.0%	
-39.00	250932	100.0%	Selected
-38.00	244796	97.6%	
-37.00	242423	96.6%	
-36.00	235541	93.9%	
-35.00	226768	90.4%	

#2:(dibr-84) Ch:1 Product

83.60	16940	6.9%	
83.70	49111	20.1%	
83.80	109442	44.8%	
83.90	197710	80.9%	
84.00	234099	95.8%	
84.10	244278	100.0%	Selected
84.20	239828	98.2%	
84.30	207285	84.9%	

84.40	165528	67.8%	
84.50	16018	6.6%	
84.60	1867	0.8%	

#2:(dibr-84) Ch:1 Q1PreBias

-30.00	228283	89.2%	
-29.00	224803	87.8%	
-28.00	228577	89.3%	
-27.00	214490	83.8%	
-26.00	210854	82.3%	
-25.00	217147	84.8%	
-24.00	214218	83.7%	
-23.00	227779	89.0%	
-22.00	237628	92.8%	
-21.00	241805	94.4%	
-20.00	236874	92.5%	
-19.00	226490	88.5%	
-18.00	234394	91.5%	
-17.00	252729	98.7%	
-16.00	256047	100.0%	Selected
-15.00	233190	91.1%	
-14.00	225243	88.0%	
-13.00	249674	97.5%	
-12.00	235471	92.0%	
-11.00	250426	97.8%	
-10.00	240674	94.0%	

#2:(dibr-84) Ch:1 Q3PreBias

-30.00	316131	96.2%

-29.00	309424	94.2%	
-28.00	312235	95.0%	
-27.00	299214	91.0%	
-26.00	296870	90.3%	
-25.00	303577	92.4%	
-24.00	311619	94.8%	
-23.00	306156	93.2%	
-22.00	311216	94.7%	
-21.00	319342	97.2%	
-20.00	322657	98.2%	
-19.00	316444	96.3%	
-18.00	306395	93.2%	
-17.00	327892	99.8%	
-16.00	323192	98.3%	
-15.00	328646	100.0%	Selected
-14.00	300371	91.4%	
-13.00	258510	78.7%	
-12.00	225794	68.7%	
-11.00	237302	72.2%	
-10.00	304519	92.7%	

C. General mass spectrometry settings:

**Parameter**	**Value set**
Electrode potential	4000 V
Interface temperature	300 °C
Heat block temperature	400 °C
Desolvation line temperature	250 °C
Nebulizing gas flow rate	3 L/min
Drying gas flow rate	6 L/min
Heating gas flow rate	12 L/min

Substance-specific mass spectrometry settings:

Substance	Precursor ion	Product ion	Dwell time (ms)	Q1 bias (V)	Collision energy (V)	Q3 bias (V)
Ibrutinib (target)	440.9	304.1	40	-10	-31	-15
Ibrutinib (qualifier)	440.9	138.1	40	-10	-28	-14
Dihydrodiol ibrutinib (target)	474.7	304.1	40	-13	-32	-15
Dihydrodiol ibrutinib (qualifier)	474.7	84.1	40	-16	-39	-15
$^{2}H_{5}$-ibrutinib (target)	445.9	309.1	40	-10	-31	-15
$^{2}H_{5}$-dihydrodiol ibrutinib (target)	479.7	309.1	40	-13	-32	-15
