# Supplementary material for: A High-Throughput Clinical Laboratory Methodology for the Therapeutic Monitoring of Ibrutinib and Dihydrodiol Ibrutinib
Source: Molecules. 2022 Jul 25;27(15):4766. doi: 10.3390/molecules27154766 (PMC9331678; doi:10.3390/molecules27154766)
Supplement: Supplementary file 1 [file molecules-27-04766-s001.zip › Supplementary File S2.pdf]

EGÉSZSÉGÜGYI TUDOMÁNYOS TANÁCS
TUDOMÁNYOS ÉS KUTATÁSETIKAI BIZOTTSÁG

H-1051 Budapest, Arany J. u.6-8.

Telefon: ***(06-1)-795-1197***

Fax: ***(06-1)-795-0167***

www.ett.hu

Elnök:
Dr. Schaff Zsuzsa
akadémikus, egyetemi tanár

Titkár:
Dr. Kardon Tamás
egyetemi adjunktus

Tagok:
Dr. Bodoky György
egyetemi magántanár

Dr. Demeter Judit
egyetemi tanár

Dr. Dóczi Tamás
akadémikus, egyetemi tanár

Dr. Falus András
akadémikus, egyetemi tanár

Dr. Fekete Andrea
egyetemi tanársegéd

Dr. Harsányi László
egyetemi tanár

Dr. Hegyi Péter
egyetemi tanár

Dr. Hosszúfalusi Nóra
egyetemi docens

Dr. Kakuk Péter
egyetemi adjunktus

Dr. Kovács Gábor
egyetemi docens

Dr. Kovács József
egyetemi tanár

Lengyel Anna
újságíró

Dr. Mayer Árpád
egyetemi magántanár

Dr. Muszbek Katalin
alapitványi igazgató

Dr. Nagy Zoltán
egyetemi tanár

Dr. Piffkó József
egyetemi tanár

Dr. Préda István
egyetemi tanár

Dr. Rigó János
egyetemi tanár

Dr. Rojkovich Bernadette
főorvos

Dr. Somfay Attila
egyetemi tanár

Susánszky Miklós
mérnök, főtanácsadó

Dr. Süveges Ildikó
egyetemi tanár

Dr. Szánthó András
egyetemi docens

Dr. Szuromi Szabolcs
egyetemi tanár

Dr. Törő Klára
egyetemi docens

Vártok Józsefné
egészségügyi menedzser

Dr. Verő Tamás
rabbi

45371-2/2016/EKU

Budapest, 2016. augusztus 31.

Melléklet: 1 db Határozat

ÉRTESITÉS
Dr. Alpár Donát témavezető részére

Az Egészségügyi Tudományos Tanács Tudományos és Kutatásetikai Bizottság (ETT TUKEB) "Klonális evolúció tér- és időbeli vizsgálata krónikus limfocitás leukémia személyre szabott kezeléséhez" című beadványt eljárásra bocsátotta. Az egészségügyről szóló 1997.évi CLIV. törvény és a 23/2002(V.9) EüM rendelet alapján

a bizottság a vizsgálatot engedélyezi, a szakmai-etikai engedélyt a vizsgálat megkezdéséhez kiadta.

A fenti rendelet értelmében a 20/O. pont, valamint a 235/2009.(X.20.) Korm. Rendelet 19.§ figyelembevételével kell eljárni a vizsgálat lezárásáról szóló elismerő okirat kiadásához.

Tájékoztatjuk, hogy a bizottság a fenti rendelet 20/S pontja alapján az eljárás megkezdését, a beszámoló értékelését az ETT honlapján 1 évig közzéteszi.

P.H.

EGÉSZSÉGÜGYI TUDOMÁNYOS TANÁCS Tudományos és Kutatásetikai Bizottság Budapest

Dr. Schaff Zsuzsa
akadémikus, egyetemi tanár,
az ETT TUKEB elnöke

Egészségügyi Tudományos Tanács
Tudományos és Kutatásetikai Bizottság (ETT TUKEB)
Egészségügyi Tudományos Tanács Titkárság
Budapest 1051 Arany János u. 6-8.

Ügyiratszám: **45371-2/2016/EKU**
Ügyintéző neve: **Dr. Kardon Tamás**
ETT TUKEB Titkárság vezető
Elérhetősége: **tukeb@emmi.gov.hu**
Telefon: **795-1197**
Melléklet: **1 db**

Tárgy: SE ÁOK I. Sz. Patológiai és Kísérleti Rákkutató Intézet (Üllői út 26., 1085) beavatkozással nem járó vizsgálat engedélyezésének kérelmezése tárgyában hozott határozat

HATÁROZAT

Dr. Alpár Donát részére "Klonális evolúció tér- és időbeli vizsgálata krónikus limfocitás leukémia személyre szabott kezeléséhez " című beavatkozással nem járó vizsgálat engedélyezése tárgyában benyújtott kérelmét (2016. augusztus 30-i ülésen), mint első fokú hatóság az alábbiak szerint bíráltam el.

A benyújtott beavatkozással nem járó vizsgálatra

a szakmai-etikai engedélyt megadom

a csatolt, 23/2002.(V.9.)EüM "Az emberen végzett orvostudományi kutatásokról" szóló rendelet 2. számú mellékletében felsorolt vizsgálóhelyekre.

Döntésem ellen a közlést követő 15 napon belül van helye fellebbezésnek a közigazgatási hatósági eljárás és szolgáltatás általános szabályairól szóló 2004. évi CXL. törvény 99. §-ának (1) bekezdése, valamint az emberen végzett orvostudományi kutatások, az emberi felhasználásra kerülő vizsgálati készítmények klinikai vizsgálata, valamint az emberen történő alkalmazásra szolgáló, klinikai vizsgálatra szánt orvostechnikai eszközök klinikai vizsgálata engedélyezési eljárásának szabályairól szóló 235/2009. (X. 20.) Korm. rendelet (a továbbiakban: Korm. rendelet) 17. §-ának (3) bekezdése szerint.

A fellebbezési eljárás díja az emberen végzett orvostudományi kutatásokról szóló 23/2002. (V. 9.) EüM rendelet (a továbbiakban: EüM rendelet) 20/R. §-ának (3) bekezdése szerinti igazgatási szolgáltatási díj. A fellebbezést a hatóságomhoz kell benyújtani.

INDOKOLÁS

A(z) SE ÁOK I. Sz. Patológiai és Kísérleti Rákkutató Intézet beavatkozással nem járó vizsgálat szakmai-etikai engedélyezésének kérelmezése iránt a Korm. rendelet 18. §-ának (1) bekezdése alapján kérelmet nyújtott be hatóságomhoz. A kérelmező mellékletként csatolta az EüM rendelet 20/ E. §-ának (2) bekezdése szerinti nyilatkozatot.

A kérelmet megvizsgáltam és megállapítottam, hogy a Korm. rendelet 18. §-a (2) bekezdése a) - c) pontjaiban foglaltaknak az alábbiak szerint felel meg:

18. § (2) bekezdése a)-c) pontjai: megfelelnek a rendelkező rész szerint.

Ezért a rendelkező részben foglaltak szerint döntöttem.

A fellebbezés lehetőségét a közigazgatási hatósági eljárás és szolgáltatás általános szabályairól szóló 2004. évi CXL. törvény 98. §-ának (1) bekezdése, valamint 99. §-ának (1) bekezdése szerint biztosítottam.

A fellebbezési igazgatási szolgáltatási díj mértékét az EüM rendelet 20/R. §-ának (3) bekezdése szerint állapítottam meg.

Hatóságom hatáskörét és illetékességét az egészségügyről szóló 1997. évi CLIV. törvény 164/A. §-a és a Korm. rendelet 18. §-a állapítja meg.

Felhívjuk figyelmét arra a jogszabályi kötelezettségére, mely szerint a kutatás megkezdésétől számított minden második év végén, valamint a kutatás befejezését követően 15 napon belül jelentést kell küldenie az engedélyezőnek, az etikai bizottságnak és az IKEB-nek.

Kérjük, amennyiben a kutatás nem kezdődik el, vagy idő előtt lezárásra kerül, akkor e-mailen és levélben is tájékoztassa az ETT TUKEB-et.

Budapest, 2016. augusztus 31.

P.H.

Dr. Schaff Zsuzsa
akadémikus, egyetemi tanár,
az ETT TUKEB elnöke

Kapják még:
1./ Témavezető
2./ Tudományos rektorhelyettes
3./ Irattár

Melléklet a(z)
45371- 2/2016/EKU
számú határozathoz

ÜRLAP ETT-TUKEB KUTATÁSI ENGEDÉLYHEZ

**Pályázat, projekt címe**: Klonális evolúció tér- és időbeli vizsgálata krónikus limfocitás leukémia személyreszabott kezeléséhez
**Témavezető neve**: Dr. Alpár Donát
**Támogató/szponzor neve, címe**: NKFI Hivatal, 1077 Budapest, Kéthly Anna tér 1. (K_16 pályázat, azonosító: K119950)
**Támogató/szponzor képviselője**: Gáll Zsuzsánna
**Témavezető kutatóhelye, címe**: Semmelweis Egyetem, I.sz. Patológiai és Kísérleti Rákkutató Intézet, H - 1085 Budapest, Üllői út 26.
**Témavezető munkahelyi telefonszáma**: +36(1)459-1500<br>**Mobil telefonszáma**: 06204261172<br>**FAX száma**: +36(1)317-1074<br>**E-mail címe**: alpar.donat@med.semmelweis-univ.hu
**Multicentrikus vizsgálat esetén a részt vevő kutatóhelyek felsorolása**:<br>Semmelweis Egyetem, I.sz. Patológiai és Kísérleti Rákkutató Intézet<br>(igazgató: Prof Dr Matolcsy András)<br>Semmelweis Egyetem, I.sz. Belgyógyászati Klinika, Hematológiai Osztály<br>(osztályvezető: Prof Dr Demeter Judit)<br>Semmelweis Egyetem, III.sz. Belgyógyászati Klinika, Hematológiai Osztály<br>(osztályvezető: Prof Dr Masszi Tamás)<br>Egyesített Szent István és Szent László Kórház Hematológiai és Őssejt-transzplantációs Osztály<br>(osztályvezető: Prof Dr Masszi Tamás)<br>Debreceni Egyetem, II.sz. Belgyógyászati Klinika<br>(osztályvezető: Prof Dr Illés Árpád)<br>Somogy Megyei Kaposi Mór Oktató Kórház, Hematológiai Osztály<br>(osztályvezető: Dr Egyed Miklós)

**Tervezett kutatás kezdete**: 2016.10.01.	**vége**: 2020.09.30.

Rövid kutatási terv magyarul

A krónikus limfocitás leukémia (CLL) B-sejtek klonális proliferációja nyomán alakul ki, klinikai kimenetele rendkívül változó. Emögött leginkább a betegek eltérő genetikai-, epigenetikai-, transzkriptomikai profiljai, valamint az érintett csontvelő és nyirokcsomók különféle mikrokörnyezeti hatásai állhatnak. A közelmúltban a rosszul reagáló CLL-es betegek kezeléséhez új célzott terápiákat fejlesztettek ki, úgymint a Bruton tirozin-kináz gátló ibrutinib és acalabrutinib, a foszfatidilinozitol-3 kináz gátló idelalisib és a B-sejtes limfóma 2 antagonista venetoclax. A betegek egy része azonban még e kezelések kapcsán is kedvezőtlen választ mutat, másodlagos rezisztenciát fejleszt ki és végül elhuny.

Vizsgálatunk munkahipotézisei: (*i*) a magas kockázatú CLL-es betegek leukémiái belső heterogenitást mutatnak makro- (perifériás vér, csontvelő és nyirokcsomó közötti) és mikro (az adott szövet egyes sejtpopulációi közötti) szinten; (*ii*) a szubklonális szerkezet és az ehhez kapcsolódó transzkripciós profil jellemezhető időbeli változásokat mutat a kezelés során, azaz a szelekció valamilyen szintű visszatérő mintázata felismerhető; (*iii*) rezisztencia mechanizmusokat tudunk felfedni a leukémiás genom és transzkriptum rezisztencia előtt és után történő vizsgálatával, valamint (*iv*) alternatív terápiás célpontokat tudunk azonosítani betegek számára, akik a jelenleg elérhető terápiákra nem reagálnak megfelelően.
