# Supplementary material for: A High-Throughput Clinical Laboratory Methodology for the Therapeutic Monitoring of Ibrutinib and Dihydrodiol Ibrutinib
Source: Molecules. 2022 Jul 25;27(15):4766. doi: 10.3390/molecules27154766 (PMC9331678; doi:10.3390/molecules27154766)
Supplement: Supplementary file 1 [file molecules-27-04766-s001.zip › Supplementary File S3.pdf]

Supplementary File S3: Scripts run in the R environment.

(A) Nonparametric and parametric regression algorithms for evaluating the relationship between concentrations of ibrutinib, dihydrodiol ibrutinib and the active moiety (ibrutinib+dihydrodiol ibrutinib), and the standard deviations obtained in parralel analyses

```
# By running this script you will perform regression using
unweighted linear least squares, unweighted second degree polynomial
least squares, and Theil's regression with and without the Siegel
correction. Regression plots will be created.


# additionally required library
library(ggplot2)


# construction of input data tables
data=read.csv(file.choose())  # headers in the data file should be:
                              # "conc", "sd"
data=data[1:21,]
sln=matrix(, nrow=nrow(data)-1, ncol=nrow(data)-1)
difn=matrix(, nrow=nrow(data)-1, ncol=nrow(data)-1)
ssln=matrix(, nrow=nrow(data), ncol=nrow(data))
sdifn=matrix(, nrow=nrow(data), ncol=nrow(data))


# Theil’s regression without the Siegel estimator
for (i in 1:(nrow(data)-1)) {
for (j in (i+1):nrow(data)) {
sln[i,j-1]=(data$sd[j]-data$sd[i])/(data$conc[j]-data$conc[i])
difn[i,j-1]=data$sd[j]-sln[i,j-1]*data$conc[j]
}
}


thsl=median(as.numeric(sln)[!is.na(sln)])
thint=median(data$sd-thsl*data$conc)
```

```
# Theil's regression with the Siegel estimator
for (k in 1:nrow(data)) {
for (l in 1:nrow(data)) {
    if (k==l) {
    ssln[k,l]=NA
    sdifn[k,l]=NA
    } else {
    ssln[k,l]=(data$sd[l]-data$sd[k])/(data$conc[l]-data$conc[k])
    sdifn[k,l]=(data$conc[l]*data$sd[k]-
data$conc[k]*data$sd[l])/(data$conc[l]-data$conc[k])
    }
  }
}

# slope
slist=split(ssln, row(ssln))
slist=lapply(slist, function(x) x[!is.na(x)])
ssl=lapply(slist,median)
siegsl=median(as.vector(unlist(ssl)))

# intercept
ilist=split(sdifn, row(sdifn))
ilist=lapply(ilist, function(x) x[!is.na(x)])
si=lapply(ilist,median)
siegi=median(as.vector(unlist(si)))

# calculating errors of estimates
nscalc=data$conc*thsl+thint
scalc=data$conc*siegsl+siegi
data=cbind(data,nscalc)
data=cbind(data,scalc)

# unweighted linear least squares
```

```
ols=lm(data$sd~data$conc)

olscalc=data$conc*ols$coefficients[[2]]+ols$coefficients[[1]]

data=cbind(data,olscalc)


# unweighted second degree polynomal least squares

poly2=lm(sd ~ poly(conc, 2, raw = TRUE), data = data)

poly2calc=poly2$coefficients[[1]]+data$conc*poly2$coefficients[[2]]+
(data$conc^2)*poly2$coefficients[[3]]

data=cbind(data,poly2calc)


# calculation of nssr values

ns_nssr=sum((data$nscalc-data$sd)^2/data$nscalc^2)

s_nssr=sum((data$scalc-data$sd)^2/data$scalc^2)

ols_nssr=sum((data$olscalc-data$sd)^2/data$olscalc^2)

poly2_nssr=sum((data$poly2calc-data$sd)^2/data$poly2calc^2)


nssr=c(ns_nssr,s_nssr,ols_nssr,poly2_nssr)

names(nssr)=c("Theil","Siegel","OLS","2nd-OLS")


# plotting

nosiegel=ggplot(data,aes(x=conc,y=sd))+geom_point(size=4)+geom_ablin
e(intercept=thint,slope=thsl, size=2) +

theme_bw() +

theme(axis.text = element_text(size = 20))


siegel=ggplot(data,aes(x=conc,y=sd))+geom_point(size=4)+geom_abline(
intercept=siegi,slope=siegsl, size=2) +

theme_bw() +

theme(axis.text = element_text(size = 20))


olsplot=ggplot(data,aes(x=conc,y=sd))+geom_point(size=4)+geom_abline
(intercept= ols$coefficients[[1]],slope= ols$coefficients[[2]],
size=2) +

theme_bw() +
```

```
theme(axis.text = element_text(size = 20))


# call ’nssr’ to display the results of nssr calculations; call’
nosiegel’ to display the regression plot obtained by applying
Theil’s regression without the Siegel estimator, call’ siegel’ to
display the regression plot obtained by applying Theil’s regression
with the Siegel estimator, call ’olsplot’ to display the unweighted
linear regression plot
```

(B) Noncompartmental pharmacokinetic analysis

```
# additionally required libraries
library(NonCompart)
library(ncar)

# load dataset
ncadata=read.csv(file.choose())           # columns: Subject
                                          #(’subj’), Time (’time’),
                                          # Concentrations (’ibr’,
                                          # ’dib’ and ’ibrdib’)

# define variables
drug="ibrdib"                             # drug identifier
fileid=paste0("p",subnum,"-",drug,".pdf") # output file name
dose.adm=0.636                            # administered dose in
                                          # units of mmol
form="Extravascular"                      # route of administration
doseunit="mmol"                           # unit of dose
timeunit="h"                              # time unit
cunit="nmol/L"                            # concentration unit
column=5                                  # number of column in
                                          # 'ncadata' containing
                                          # the concentrations of
```

```
                                          # the respective drug
                                          #(3: ibr, 4: dibr,
                                          # 5: ibr+dibr)

auc=AUC(ncadata$time,ncadata[,column], down="Linear")

# Perform single-subject NCA and make report
pdfNCA(fileName=fileid,ncadata,key="subj",colTime="time",colConc=drug,dose=dose.adm,adm=form,dur=0,doseUnit=doseunit,timeUnit=timeunit,concUnit=cunit,down="Linear",R2ADJ=0,MW=0,iAUC="",excludeDelta=0.3)
write.csv(auc,file=paste0("auc-p",subnum,"-",drug,".csv"))
```
