# Supplementary material for: A High-Throughput Clinical Laboratory Methodology for the Therapeutic Monitoring of Ibrutinib and Dihydrodiol Ibrutinib
Source: Molecules. 2022 Jul 25;27(15):4766. doi: 10.3390/molecules27154766 (PMC9331678; doi:10.3390/molecules27154766)

Supplementary File S4: Representative ion chromatograms of ibrutinib (IBR), dihydrodiol ibrutinib (DIB), and their respective internal standards $^{2}H_{5}$-ibrutinib ($^{2}H_{5}$-IBR) and $^{2}H_{5}$-dihydrodiol ibrutinib ($^{2}H_{5}$-DIB). (A), IBR in the level 1 calibration sample (2.27 nmol/L), (B) $^{2}H_{5}$-IBR in the level 1 calibration sample, (C) IBR in a blank sample, (D) $^{2}H_{5}$-IBR in a blank sample, (E) DIB in the level 1 calibration sample (2.11 nmol/L), (F) $^{2}H_{5}$-DIB in the level 1 calibration sample, (G) DIB in a blank sample, (H) $^{2}H_{5}$-DIB in a blank sample.

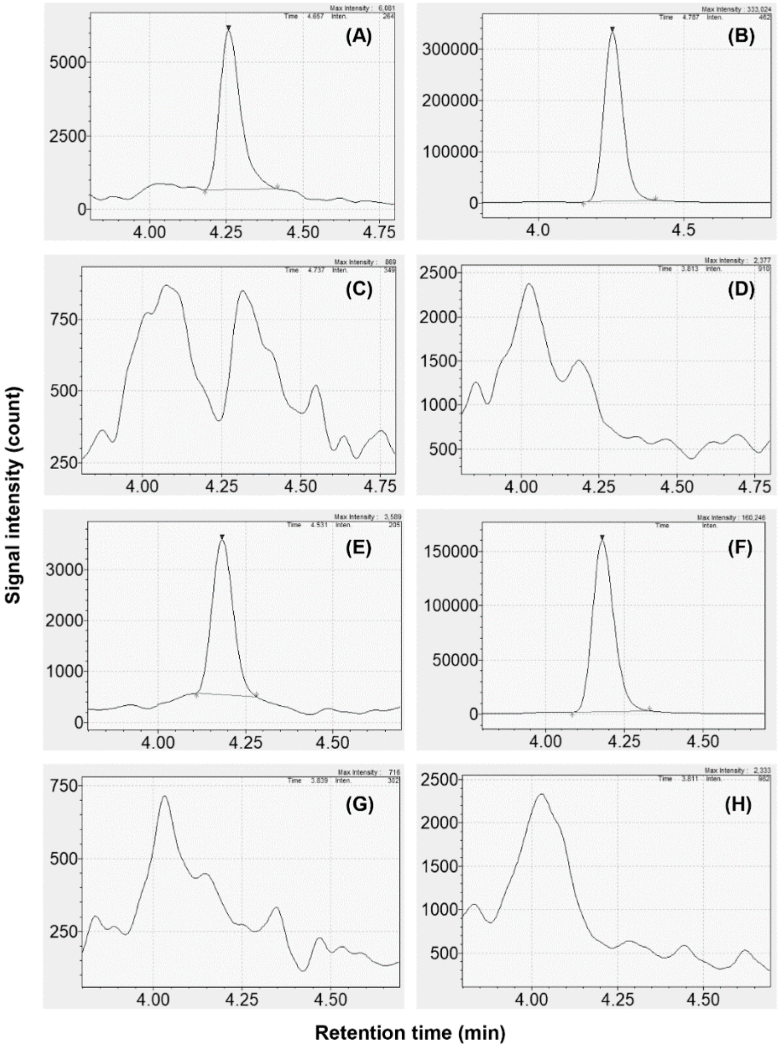

Supplement: Supplementary file 1 [file molecules-27-04766-s001.zip › Supplementary File S4.pdf]
