# Supplementary material for: A High-Throughput Clinical Laboratory Methodology for the Therapeutic Monitoring of Ibrutinib and Dihydrodiol Ibrutinib
Source: Molecules. 2022 Jul 25;27(15):4766. doi: 10.3390/molecules27154766 (PMC9331678; doi:10.3390/molecules27154766)
Supplement: Supplementary file 1 [file molecules-27-04766-s001.zip › Supplementary File S5.pdf]

Individual Noncompartmental Analysis Result

subj=1

NONCOMPARTMENTAL ANALYSIS REPORT
Package version 0.4.5 (2022−02−23)
R version 4.0.5 (2021−03−31)

Date and Time: 2022−04−01 22:04:24 Europe/Prague

Calculation Setting

Drug Administration: Extravascular
Observation count excluding trailing zero: 7
Dose at time 0: 0.953 mmol
AUC Calculation Method: Linear−up Linear−down
Weighting for lambda z: Uniform (Ordinary Least Square, OLS)
Lambda z selection criterion: Heighest adjusted R−squared value with precision=1e−4

Fitting, AUC, AUMC Result

Time		Conc.	Pred.	Residual	AUC	AUMC
0.0000	+	0.0000			0.0000	0.0000
0.0100		8.2000			0.0410	0.0004
0.5000		68.4000			18.8080	8.3995
1.0000		250.9000			98.6330	79.6745
2.0000		265.6000			356.8830	470.7245
4.0000	*	101.7000	101.6711	+2.889e−02	724.1830	1408.7245
23.0000	*	9.2000	9.2524	−5.243e−02	1777.7330	7283.5245
24.0000	*	8.2000	8.1559	+4.415e−02	1786.4330	7487.7245

+: Back extrapolated concentration
*: Used for the calculation of Lambda z.

Calculated Values

CMAX	Max Conc	265.6000	nmol/L
CMAXD	Max Conc Norm by Dose	278.6988	nmol/L/mmol
TMAX	Time of CMAX	2.0000	h
TLAG	Time Until First Nonzero Conc	0.0000	h
CLST	Last Nonzero Conc	8.2000	nmol/L
CLSTP	Last Nonzero Conc Pred	8.1559	nmol/L
TLST	Time of Last Nonzero Conc	24.0000	h
LAMZHL	Half−Life Lambda z	5.4946	h
LAMZ	Lambda z	0.1262	/h
LAMZLL	Lambda z Lower Limit	4.0000	h
LAMZUL	Lambda z Upper Limit	24.0000	h
LAMZNPT	Number of Points for Lambda z	3	
CORRXY	Correlation Between TimeX and Log ConcY	−1.0000	
R2	R Squared	1.0000	
R2ADJ	R Squared Adjusted	1.0000	
AUCLST	AUC to Last Nonzero Conc	1786.4330	h*nmol/L
AUCALL	AUC All	1786.4330	h*nmol/L
AUCIFO	AUC Infinity Obs	1851.4348	h*nmol/L
AUCIFOD	AUC Infinity Obs Norm by Dose	1942.7437	h*nmol/L/mmol

subj=1

AUCIFP	AUC Infinity Pred	1851.0848	h*nmol/L
AUCIFPD	AUC Infinity Pred Norm by Dose	1942.3765	h*nmol/L/mmol
AUCPEO	AUC %Extrapolation Obs	3.5109	%
AUCPEP	AUC %Extrapolation Pred	3.4926	%
AUMCLST	AUMC to Last Nonzero Conc	7487.7245	h2*nmol/L
AUMCIFO	AUMC Infinity Obs	9563.0397	h2*nmol/L
AUMCIFP	AUMC Infinity Pred	9551.8664	h2*nmol/L
AUMCPEO	AUMC %Extrapolation Obs	21.7014	%
AUMCPEP	AUMC % Extrapolation Pred	21.6098	%
VZFO	Vz Obs by F	4080.3361	L
VZFP	Vz Pred by F	4081.1075	L
CLFO	Total CL Obs by F	514.7359	L/h
CLFP	Total CL Pred by F	514.8332	L/h
MRTEVLST	MRT Extravasc to Last Nonzero Conc	4.1914	h
MRTEVIFO	MRT Extravasc Infinity Obs	5.1652	h
MRTEVIFP	MRT Extravasc Infinity Pred	5.1601	h

subj=1

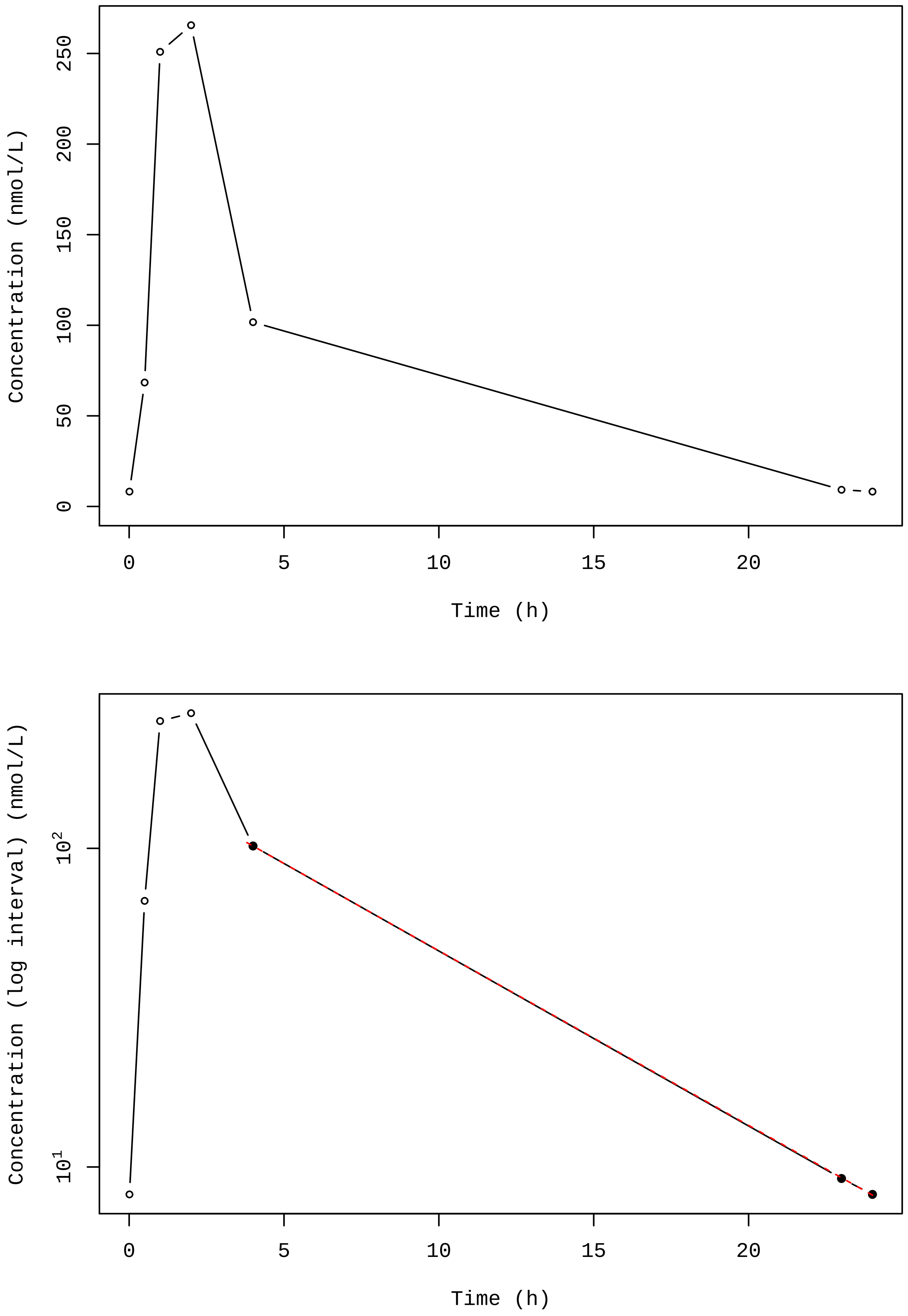

Concentration (nmol/L)
0
50
100
150
200
250
0
5
10
15
20
Time (h)
Concentration (log interval) (nmol/L)
10^1
10^2
0
5
10
15
20
Time (h)

Individual Noncompartmental Analysis Result

subj=1

NONCOMPARTMENTAL ANALYSIS REPORT
Package version 0.4.5 (2022−02−23)
R version 4.0.5 (2021−03−31)

Date and Time: 2022−07−12 22:39:35 Europe/Prague

Calculation Setting

Drug Administration: Extravascular
Observation count excluding trailing zero: 7
Dose at time 0: 0 mmol
AUC Calculation Method: Linear−up Linear−down
Weighting for lambda z: Uniform (Ordinary Least Square, OLS)
Lambda z selection criterion: Heighest adjusted R−squared value with precision=1e−4

Fitting, AUC, AUMC Result

Time		Conc.	Pred.	Residual	AUC	AUMC
0.0000	+	0.0000			0.0000	0.0000
0.0100		34.6322			0.1732	0.0017
0.5000		53.3565			21.7304	6.6228
1.0000		139.0137			69.8230	48.0457
2.0000		184.7313			231.6955	302.2839
4.0000	*	142.9294	143.3264	−3.970e−01	559.3562	1243.4641
23.0000	*	41.3200	39.0901	+2.230e+00	2309.7253	15703.1982
24.0000	*	34.6322	36.5064	−1.874e+00	2347.7014	16593.9650

+: Back extrapolated concentration
*: Used for the calculation of Lambda z.

Calculated Values

CMAX	Max Conc	184.7313	nmol/L
TMAX	Time of CMAX	2.0000	h
TLAG	Time Until First Nonzero Conc	0.0000	h
CLST	Last Nonzero Conc	34.6322	nmol/L
CLSTP	Last Nonzero Conc Pred	36.5064	nmol/L
TLST	Time of Last Nonzero Conc	24.0000	h
LAMZHL	Half−Life Lambda z	10.1364	h
LAMZ	Lambda z	0.0684	/h
LAMZLL	Lambda z Lower Limit	4.0000	h
LAMZUL	Lambda z Upper Limit	24.0000	h
LAMZNPT	Number of Points for Lambda z	3	
CORRXY	Correlation Between TimeX and Log ConcY	−0.9975	
R2	R Squared	0.9951	
R2ADJ	R Squared Adjusted	0.9902	
AUCLST	AUC to Last Nonzero Conc	2347.7014	h*nmol/L
AUCALL	AUC All	2347.7014	h*nmol/L
AUCIFO	AUC Infinity Obs	2854.1553	h*nmol/L
AUCIFP	AUC Infinity Pred	2881.5627	h*nmol/L
AUCPEO	AUC %Extrapolation Obs	17.7444	%

subj=1

AUCPEP	AUC %Extrapolation Pred	18.5268	%
AUMCLST	AUMC to Last Nonzero Conc	16593.9650	h2*nmol/L
AUMCIFO	AUMC Infinity Obs	36155.1204	h2*nmol/L
AUMCIFP	AUMC Infinity Pred	37213.6967	h2*nmol/L
AUMCPEO	AUMC %Extrapolation Obs	54.1034	%
AUMCPEP	AUMC % Extrapolation Pred	55.4090	%
VZFO	Vz Obs by F	0.0000	L
VZFP	Vz Pred by F	0.0000	L
CLFO	Total CL Obs by F	0.0000	L/h
CLFP	Total CL Pred by F	0.0000	L/h
MRTEVLST	MRT Extravasc to Last Nonzero Conc	7.0682	h
MRTEVIFO	MRT Extravasc Infinity Obs	12.6675	h
MRTEVIFP	MRT Extravasc Infinity Pred	12.9144	h

subj=1

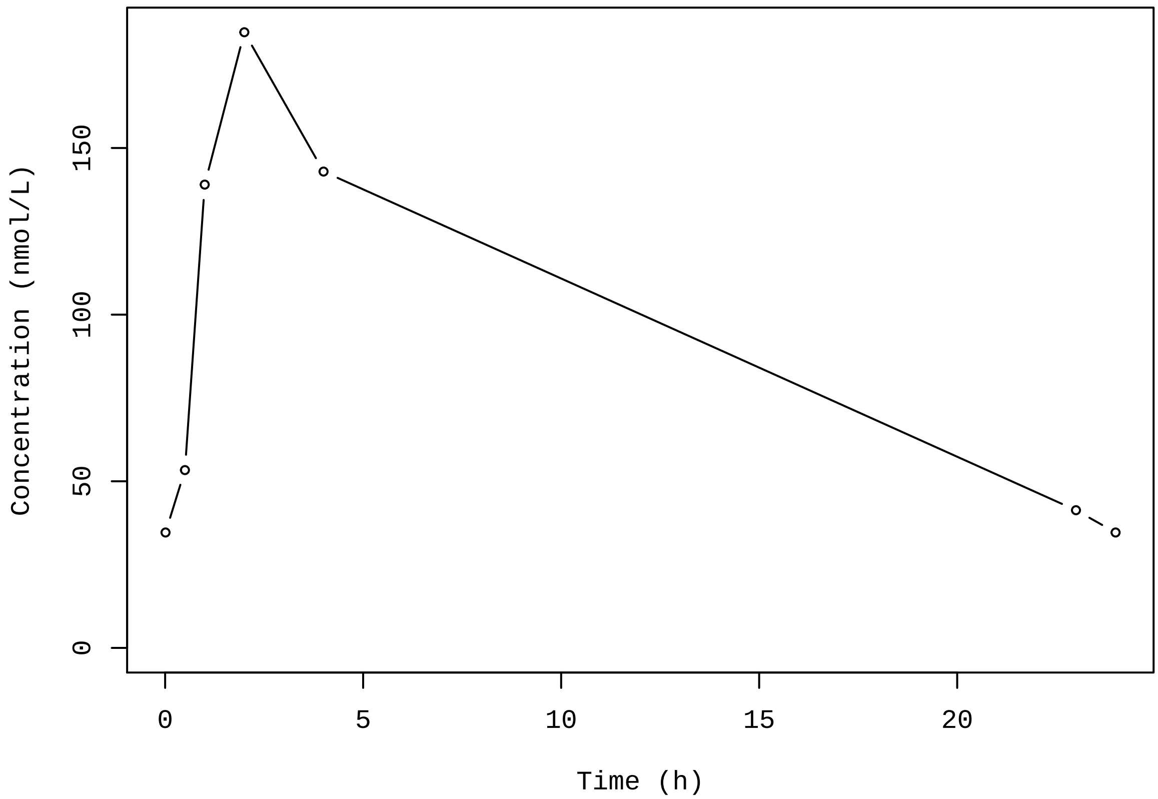

Concentration (nmol/L)
0
50
100
150
0
5
10
15
20
Time (h)

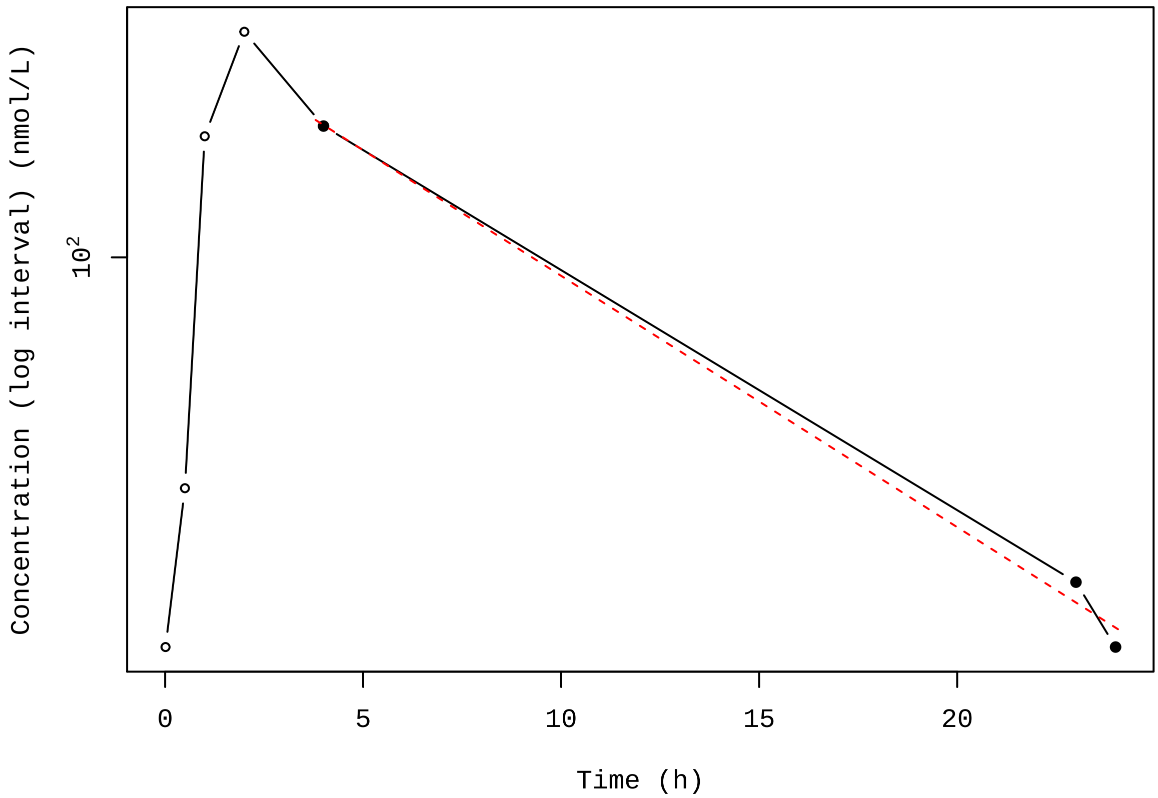

Concentration (log interval) (nmol/L)
10^2
0
5
10
15
20
Time (h)

Individual Noncompartmental Analysis Result

subj=1

NONCOMPARTMENTAL ANALYSIS REPORT
Package version 0.4.5 (2022−02−23)
R version 4.0.5 (2021−03−31)

Date and Time: 2022−04−01 22:09:18 Europe/Prague

Calculation Setting

Drug Administration: Extravascular
Observation count excluding trailing zero: 7
Dose at time 0: 0.953 mmol
AUC Calculation Method: Linear−up Linear−down
Weighting for lambda z: Uniform (Ordinary Least Square, OLS)
Lambda z selection criterion: Heighest adjusted R−squared value with precision=1e−4

Fitting, AUC, AUMC Result

Time		Conc.	Pred.	Residual	AUC	AUMC
0.0000	+	0.0000			0.0000	0.0000
0.0100		42.8322			0.2142	0.0021
0.5000		121.7565			40.5384	15.0223
1.0000		389.9137			168.4560	127.7202
2.0000		450.3313			588.5785	773.0084
4.0000	*	244.6294	245.1304	−5.010e−01	1283.5392	2652.1886
23.0000	*	50.5200	48.4944	+2.026e+00	4087.4583	22986.7227
24.0000	*	42.8322	44.5301	−1.698e+00	4134.1344	24081.6895

+: Back extrapolated concentration
*: Used for the calculation of Lambda z.

Calculated Values

CMAX	Max Conc	450.3313	nmol/L
CMAXD	Max Conc Norm by Dose	472.5407	nmol/L/mmol
TMAX	Time of CMAX	2.0000	h
TLAG	Time Until First Nonzero Conc	0.0000	h
CLST	Last Nonzero Conc	42.8322	nmol/L
CLSTP	Last Nonzero Conc Pred	44.5301	nmol/L
TLST	Time of Last Nonzero Conc	24.0000	h
LAMZHL	Half−Life Lambda z	8.1278	h
LAMZ	Lambda z	0.0853	/h
LAMZLL	Lambda z Lower Limit	4.0000	h
LAMZUL	Lambda z Upper Limit	24.0000	h
LAMZNPT	Number of Points for Lambda z	3	
CORRXY	Correlation Between TimeX and Log ConcY	−0.9991	
R2	R Squared	0.9983	
R2ADJ	R Squared Adjusted	0.9966	
AUCLST	AUC to Last Nonzero Conc	4134.1344	h*nmol/L
AUCALL	AUC All	4134.1344	h*nmol/L
AUCIFO	AUC Infinity Obs	4636.3816	h*nmol/L
AUCIFOD	AUC Infinity Obs Norm by Dose	4865.0384	h*nmol/L/mmol

subj=1

AUCIFP	AUC Infinity Pred	4656.2911	h*nmol/L
AUCIFPD	AUC Infinity Pred Norm by Dose	4885.9298	h*nmol/L/mmol
AUCPEO	AUC %Extrapolation Obs	10.8327	%
AUCPEP	AUC %Extrapolation Pred	11.2140	%
AUMCLST	AUMC to Last Nonzero Conc	24081.6895	h2*nmol/L
AUMCIFO	AUMC Infinity Obs	42024.9298	h2*nmol/L
AUMCIFP	AUMC Infinity Pred	42736.2144	h2*nmol/L
AUMCPEO	AUMC %Extrapolation Obs	42.6967	%
AUMCPEP	AUMC % Extrapolation Pred	43.6504	%
VZFO	Vz Obs by F	2410.2407	L
VZFP	Vz Pred by F	2399.9350	L
CLFO	Total CL Obs by F	205.5482	L/h
CLFP	Total CL Pred by F	204.6693	L/h
MRTEVLST	MRT Extravasc to Last Nonzero Conc	5.8251	h
MRTEVIFO	MRT Extravasc Infinity Obs	9.0642	h
MRTEVIFP	MRT Extravasc Infinity Pred	9.1782	h

subj=1

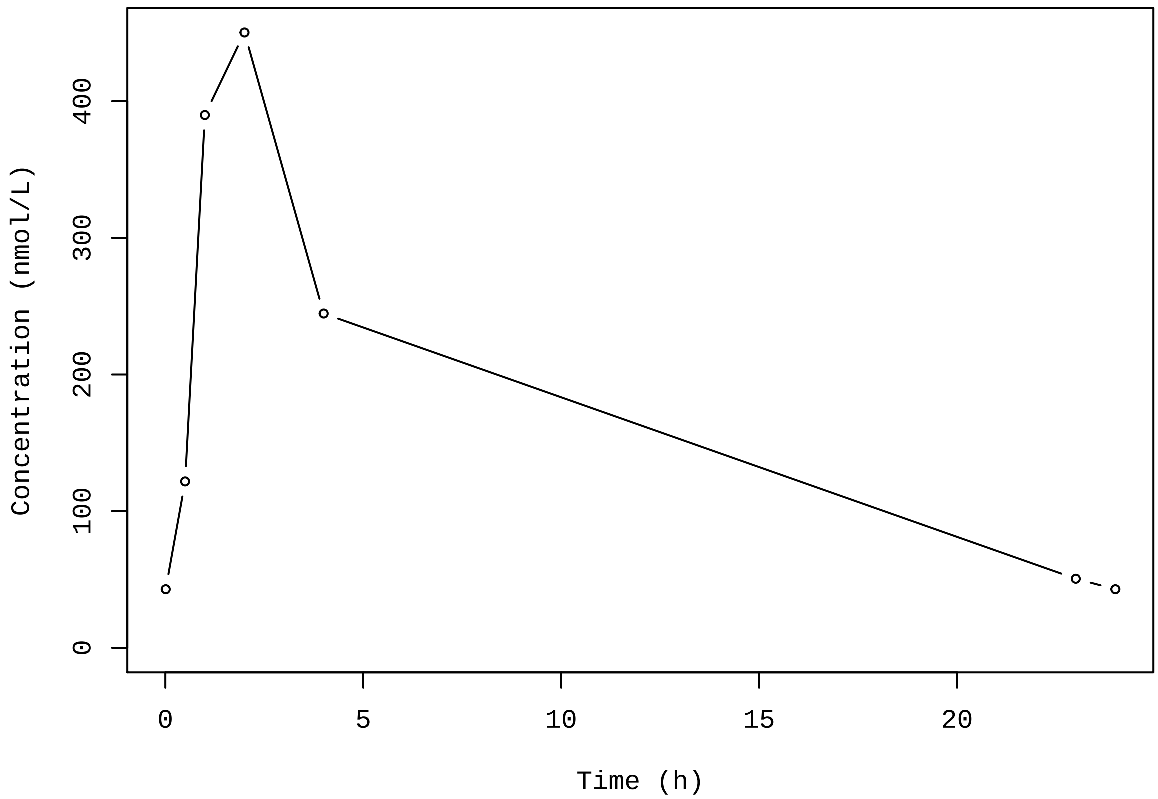

Concentration (nmol/L)
0
100
200
300
400
0
5
10
15
20
Time (h)

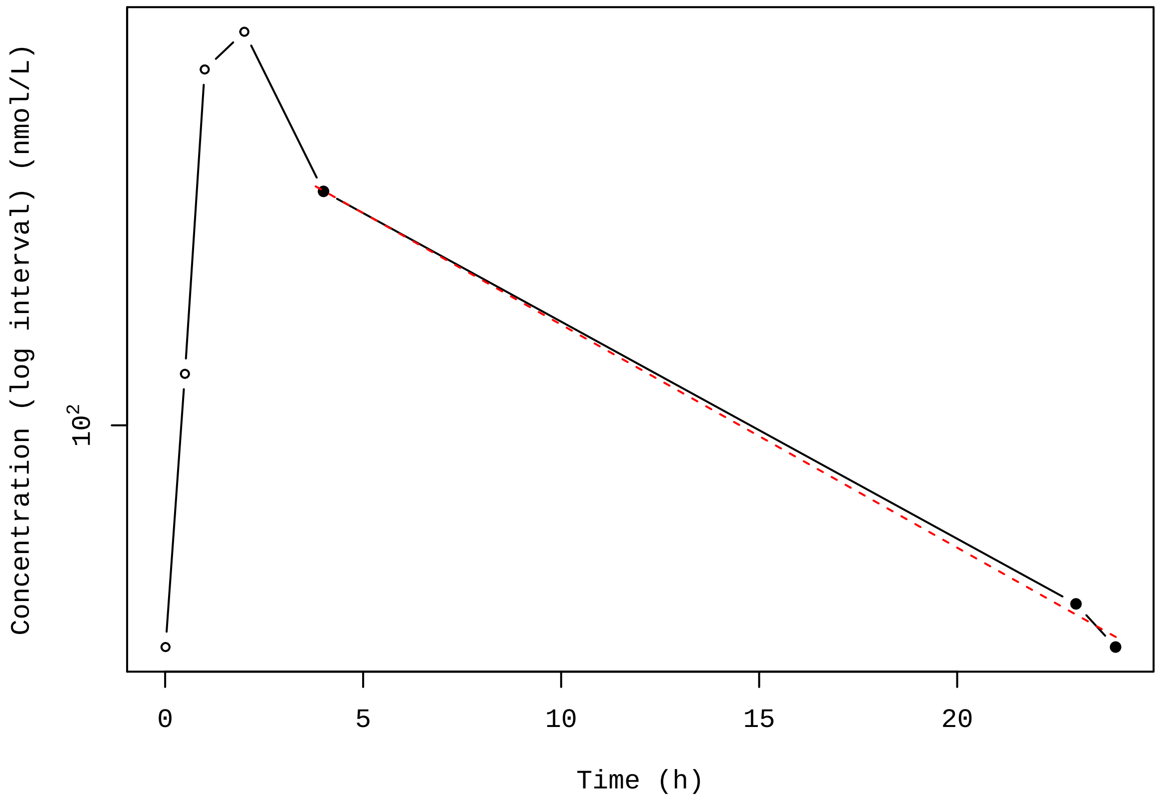

Concentration (log interval) (nmol/L)
10^2
0
5
10
15
20
Time (h)

Individual Noncompartmental Analysis Result

subj=2

NONCOMPARTMENTAL ANALYSIS REPORT
Package version 0.4.5 (2022−02−23)
R version 4.0.5 (2021−03−31)

Date and Time: 2022−04−02 23:29:02 Europe/Prague

Calculation Setting

Drug Administration: Extravascular
Observation count excluding trailing zero: 7
Dose at time 0: 0.953 mmol
AUC Calculation Method: Linear−up Linear−down
Weighting for lambda z: Uniform (Ordinary Least Square, OLS)
Lambda z selection criterion: Heighest adjusted R−squared value with precision=1e−4

Fitting, AUC, AUMC Result

Time		Conc.	Pred.	Residual	AUC	AUMC
0.0000	+	0.0000			0.0000	0.0000
0.0100		9.3000			0.0465	0.0005
0.5000		173.3000			44.7835	21.2525
1.0000		374.0000			181.6085	136.4150
2.0000		191.5000			464.3585	514.9150
4.0000	*	92.2000	92.3507	−1.507e−01	748.0585	1266.7150
23.0000	*	11.1000	10.7432	+3.568e−01	1729.4085	7195.6650
24.0000	*	9.3000	9.5932	−2.932e−01	1739.6085	7434.9150

+: Back extrapolated concentration
*: Used for the calculation of Lambda z.

Calculated Values

CMAX	Max Conc	374.0000	nmol/L
CMAXD	Max Conc Norm by Dose	392.4449	nmol/L/mmol
TMAX	Time of CMAX	1.0000	h
TLAG	Time Until First Nonzero Conc	0.0000	h
CLST	Last Nonzero Conc	9.3000	nmol/L
CLSTP	Last Nonzero Conc Pred	9.5932	nmol/L
TLST	Time of Last Nonzero Conc	24.0000	h
LAMZHL	Half−Life Lambda z	6.1217	h
LAMZ	Lambda z	0.1132	/h
LAMZLL	Lambda z Lower Limit	4.0000	h
LAMZUL	Lambda z Upper Limit	24.0000	h
LAMZNPT	Number of Points for Lambda z	3	
CORRXY	Correlation Between TimeX and Log ConcY	−0.9997	
R2	R Squared	0.9994	
R2ADJ	R Squared Adjusted	0.9988	
AUCLST	AUC to Last Nonzero Conc	1739.6085	h*nmol/L
AUCALL	AUC All	1739.6085	h*nmol/L
AUCIFO	AUC Infinity Obs	1821.7442	h*nmol/L
AUCIFOD	AUC Infinity Obs Norm by Dose	1911.5889	h*nmol/L/mmol

subj=2

AUCIFP	AUC Infinity Pred	1824.3333	h*nmol/L
AUCIFPD	AUC Infinity Pred Norm by Dose	1914.3057	h*nmol/L/mmol
AUCPEO	AUC %Extrapolation Obs	4.5086	%
AUCPEP	AUC %Extrapolation Pred	4.6442	%
AUMCLST	AUMC to Last Nonzero Conc	7434.9150	h2*nmol/L
AUMCIFO	AUMC Infinity Obs	10131.5791	h2*nmol/L
AUMCIFP	AUMC Infinity Pred	10216.5824	h2*nmol/L
AUMCPEO	AUMC %Extrapolation Obs	26.6164	%
AUMCPEP	AUMC % Extrapolation Pred	27.2270	%
VZFO	Vz Obs by F	4620.1354	L
VZFP	Vz Pred by F	4613.5786	L
CLFO	Total CL Obs by F	523.1250	L/h
CLFP	Total CL Pred by F	522.3826	L/h
MRTEVLST	MRT Extravasc to Last Nonzero Conc	4.2739	h
MRTEVIFO	MRT Extravasc Infinity Obs	5.5615	h
MRTEVIFP	MRT Extravasc Infinity Pred	5.6002	h

subj=2

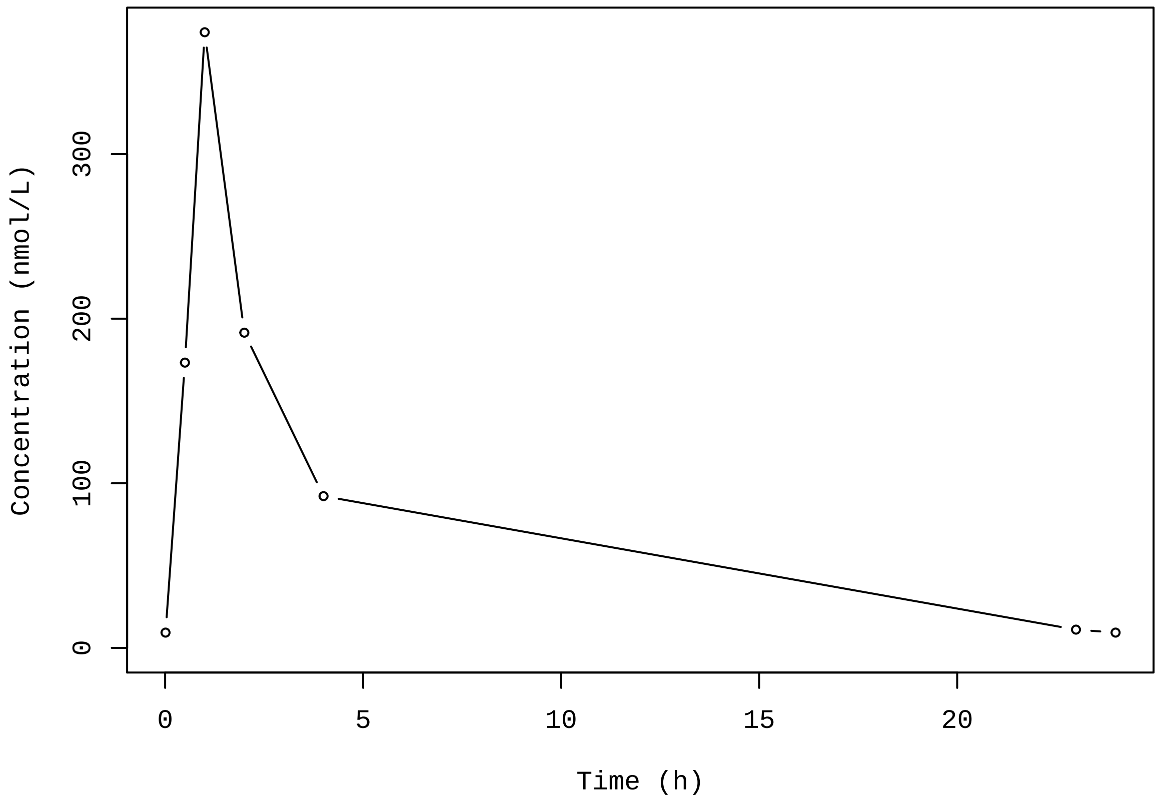

Concentration (nmol/L)
0
100
200
300
0
5
10
15
20
Time (h)

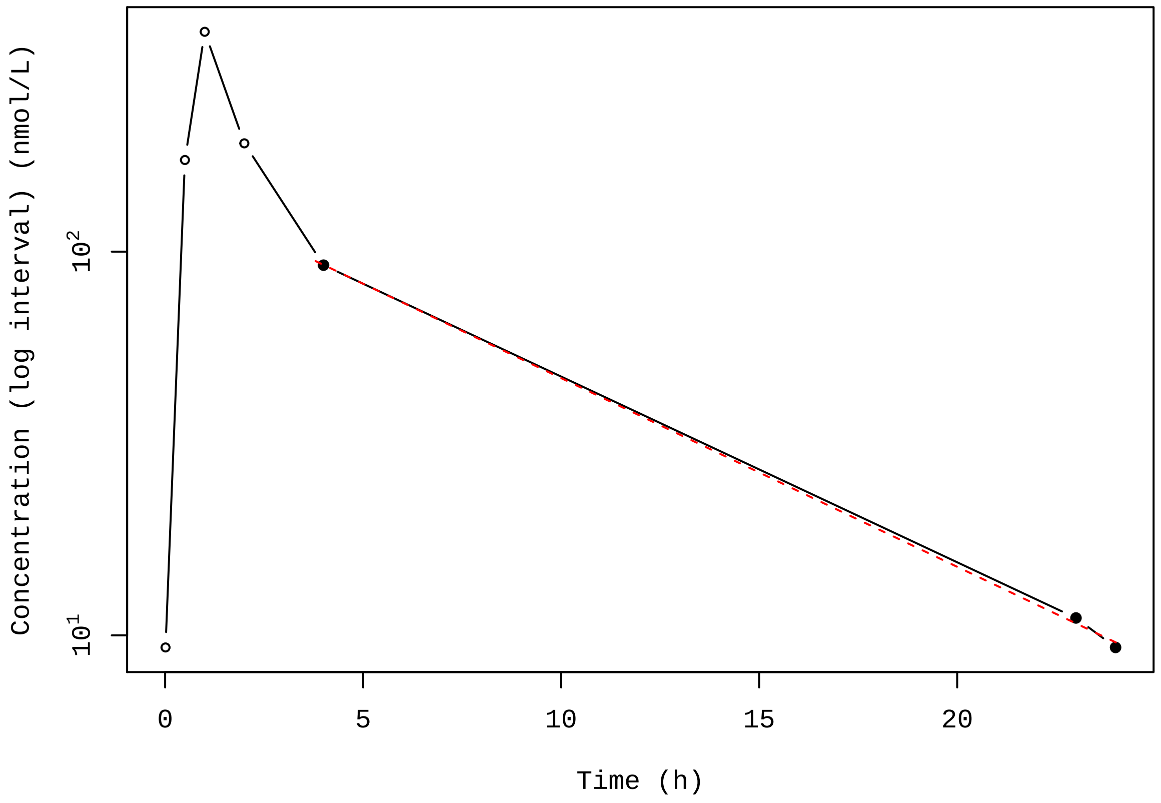

Concentration (log interval) (nmol/L)
10^1
10^2
0
5
10
15
20
Time (h)

Individual Noncompartmental Analysis Result

subj=2

NONCOMPARTMENTAL ANALYSIS REPORT
Package version 0.4.5 (2022−02−23)
R version 4.0.5 (2021−03−31)

Date and Time: 2022−07−12 22:39:07 Europe/Prague

Calculation Setting

Drug Administration: Extravascular
Observation count excluding trailing zero: 7
Dose at time 0: 0 mmol
AUC Calculation Method: Linear−up Linear−down
Weighting for lambda z: Uniform (Ordinary Least Square, OLS)
Lambda z selection criterion: Heighest adjusted R−squared value with precision=1e−4

Fitting, AUC, AUMC Result

Time		Conc.	Pred.	Residual	AUC	AUMC
0.0000	+	0.0000			0.0000	0.0000
0.0100		13.1907			0.0660	0.0007
0.5000		71.5033			20.8160	8.7921
1.0000		253.7014			102.1172	81.1554
2.0000	*	227.1106	224.9858	+2.125e+00	342.5232	435.1168
4.0000	*	171.9543	173.6181	−1.664e+00	741.5882	1577.1554
23.0000	*	14.5915	14.8004	−2.089e−01	2513.7737	11299.6630
24.0000	*	13.1907	13.0015	+1.892e−01	2527.6648	11625.7539

+: Back extrapolated concentration
*: Used for the calculation of Lambda z.

Calculated Values

CMAX	Max Conc	253.7014	nmol/L
TMAX	Time of CMAX	1.0000	h
TLAG	Time Until First Nonzero Conc	0.0000	h
CLST	Last Nonzero Conc	13.1907	nmol/L
CLSTP	Last Nonzero Conc Pred	13.0015	nmol/L
TLST	Time of Last Nonzero Conc	24.0000	h
LAMZHL	Half−Life Lambda z	5.3488	h
LAMZ	Lambda z	0.1296	/h
LAMZLL	Lambda z Lower Limit	2.0000	h
LAMZUL	Lambda z Upper Limit	24.0000	h
LAMZNPT	Number of Points for Lambda z	4	
CORRXY	Correlation Between TimeX and Log ConcY	−1.0000	
R2	R Squared	0.9999	
R2ADJ	R Squared Adjusted	0.9999	
AUCLST	AUC to Last Nonzero Conc	2527.6648	h*nmol/L
AUCALL	AUC All	2527.6648	h*nmol/L
AUCIFO	AUC Infinity Obs	2629.4533	h*nmol/L
AUCIFP	AUC Infinity Pred	2627.9934	h*nmol/L
AUCPEO	AUC %Extrapolation Obs	3.8711	%

subj=2

AUCPEP	AUC %Extrapolation Pred	3.8177	%
AUMCLST	AUMC to Last Nonzero Conc	11625.7539	h2*nmol/L
AUMCIFO	AUMC Infinity Obs	14854.1469	h2*nmol/L
AUMCIFP	AUMC Infinity Pred	14807.8422	h2*nmol/L
AUMCPEO	AUMC %Extrapolation Obs	21.7340	%
AUMCPEP	AUMC % Extrapolation Pred	21.4892	%
VZFO	Vz Obs by F	0.0000	L
VZFP	Vz Pred by F	0.0000	L
CLFO	Total CL Obs by F	0.0000	L/h
CLFP	Total CL Pred by F	0.0000	L/h
MRTEVLST	MRT Extravasc to Last Nonzero Conc	4.5994	h
MRTEVIFO	MRT Extravasc Infinity Obs	5.6491	h
MRTEVIFP	MRT Extravasc Infinity Pred	5.6347	h

subj=2

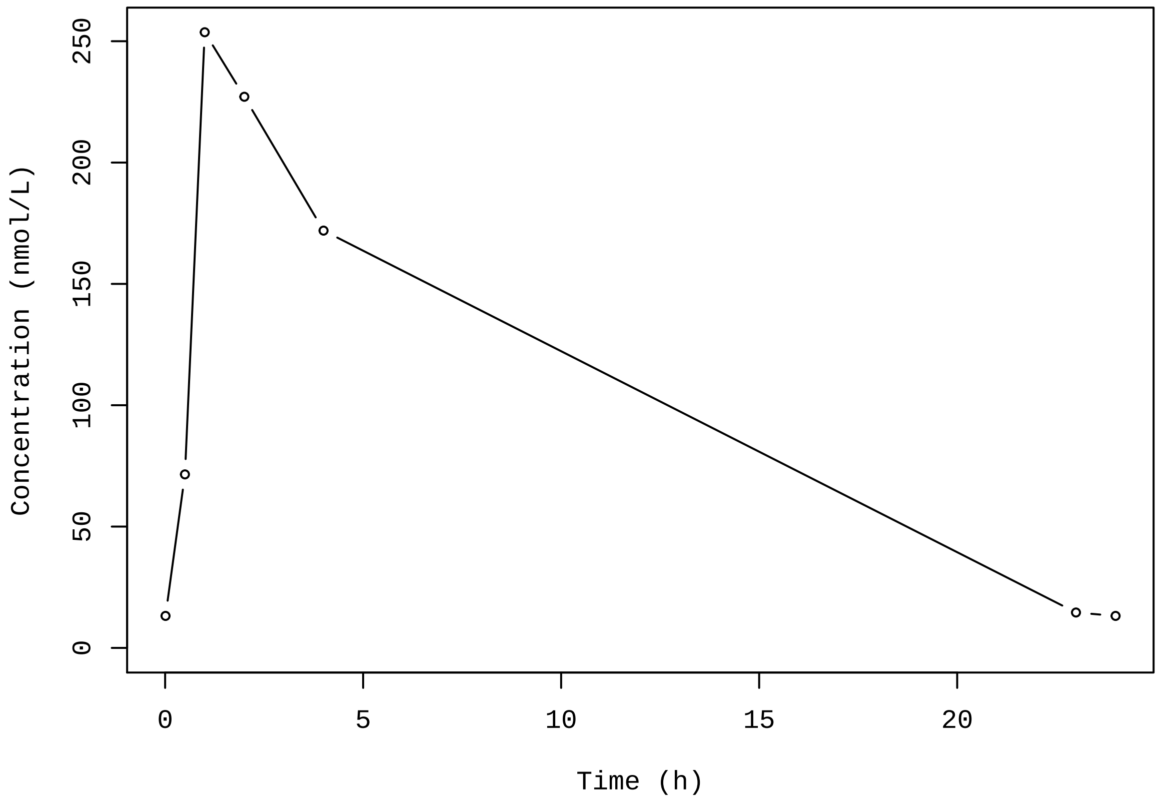

Concentration (nmol/L)
0
50
100
150
200
250
0
5
10
15
20
Time (h)

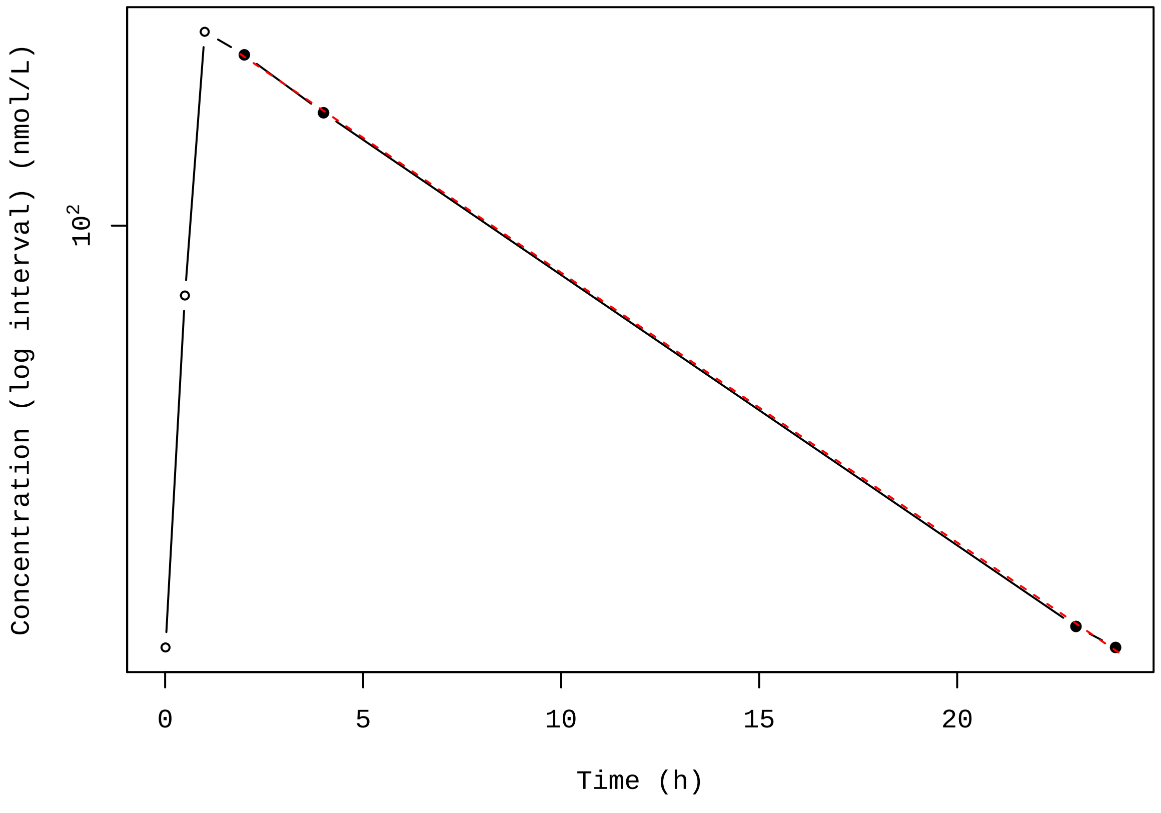

Concentration (log interval) (nmol/L)
10^2
0
5
10
15
20
Time (h)

Individual Noncompartmental Analysis Result

subj=2

NONCOMPARTMENTAL ANALYSIS REPORT
Package version 0.4.5 (2022−02−23)
R version 4.0.5 (2021−03−31)

Date and Time: 2022−04−02 23:30:33 Europe/Prague

Calculation Setting

Drug Administration: Extravascular
Observation count excluding trailing zero: 7
Dose at time 0: 0.953 mmol
AUC Calculation Method: Linear−up Linear−down
Weighting for lambda z: Uniform (Ordinary Least Square, OLS)
Lambda z selection criterion: Heighest adjusted R−squared value with precision=1e−4

Fitting, AUC, AUMC Result

Time		Conc.	Pred.	Residual	AUC	AUMC
0.0000	+	0.0000			0.0000	0.0000
0.0100		22.5407			0.1127	0.0011
0.5000		244.8533			65.6242	30.0509
1.0000		627.6727			283.7557	217.5757
2.0000		418.5968			806.8905	950.0089
4.0000	*	264.1571	264.2219	−6.481e−02	1489.6445	2843.8311
23.0000	*	25.7319	25.6059	+1.259e−01	4243.5900	18504.2161
24.0000	*	22.5407	22.6460	−1.053e−01	4267.7263	19070.6211

+: Back extrapolated concentration
*: Used for the calculation of Lambda z.

Calculated Values

CMAX	Max Conc	627.6727	nmol/L
CMAXD	Max Conc Norm by Dose	658.6282	nmol/L/mmol
TMAX	Time of CMAX	1.0000	h
TLAG	Time Until First Nonzero Conc	0.0000	h
CLST	Last Nonzero Conc	22.5407	nmol/L
CLSTP	Last Nonzero Conc Pred	22.6460	nmol/L
TLST	Time of Last Nonzero Conc	24.0000	h
LAMZHL	Half−Life Lambda z	5.6427	h
LAMZ	Lambda z	0.1228	/h
LAMZLL	Lambda z Lower Limit	4.0000	h
LAMZUL	Lambda z Upper Limit	24.0000	h
LAMZNPT	Number of Points for Lambda z	3	
CORRXY	Correlation Between TimeX and Log ConcY	−1.0000	
R2	R Squared	1.0000	
R2ADJ	R Squared Adjusted	1.0000	
AUCLST	AUC to Last Nonzero Conc	4267.7263	h*nmol/L
AUCALL	AUC All	4267.7263	h*nmol/L
AUCIFO	AUC Infinity Obs	4451.2224	h*nmol/L
AUCIFOD	AUC Infinity Obs Norm by Dose	4670.7475	h*nmol/L/mmol

subj=2

AUCIFP	AUC Infinity Pred	4452.0797	h*nmol/L
AUCIFPD	AUC Infinity Pred Norm by Dose	4671.6471	h*nmol/L/mmol
AUCPEO	AUC %Extrapolation Obs	4.1224	%
AUCPEP	AUC %Extrapolation Pred	4.1408	%
AUMCLST	AUMC to Last Nonzero Conc	19070.6211	h2*nmol/L
AUMCIFO	AUMC Infinity Obs	24968.3043	h2*nmol/L
AUMCIFP	AUMC Infinity Pred	24995.8570	h2*nmol/L
AUMCPEO	AUMC %Extrapolation Obs	23.6207	%
AUMCPEP	AUMC % Extrapolation Pred	23.7049	%
VZFO	Vz Obs by F	1742.9013	L
VZFP	Vz Pred by F	1742.5657	L
CLFO	Total CL Obs by F	214.0985	L/h
CLFP	Total CL Pred by F	214.0573	L/h
MRTEVLST	MRT Extravasc to Last Nonzero Conc	4.4686	h
MRTEVIFO	MRT Extravasc Infinity Obs	5.6093	h
MRTEVIFP	MRT Extravasc Infinity Pred	5.6144	h

subj=2

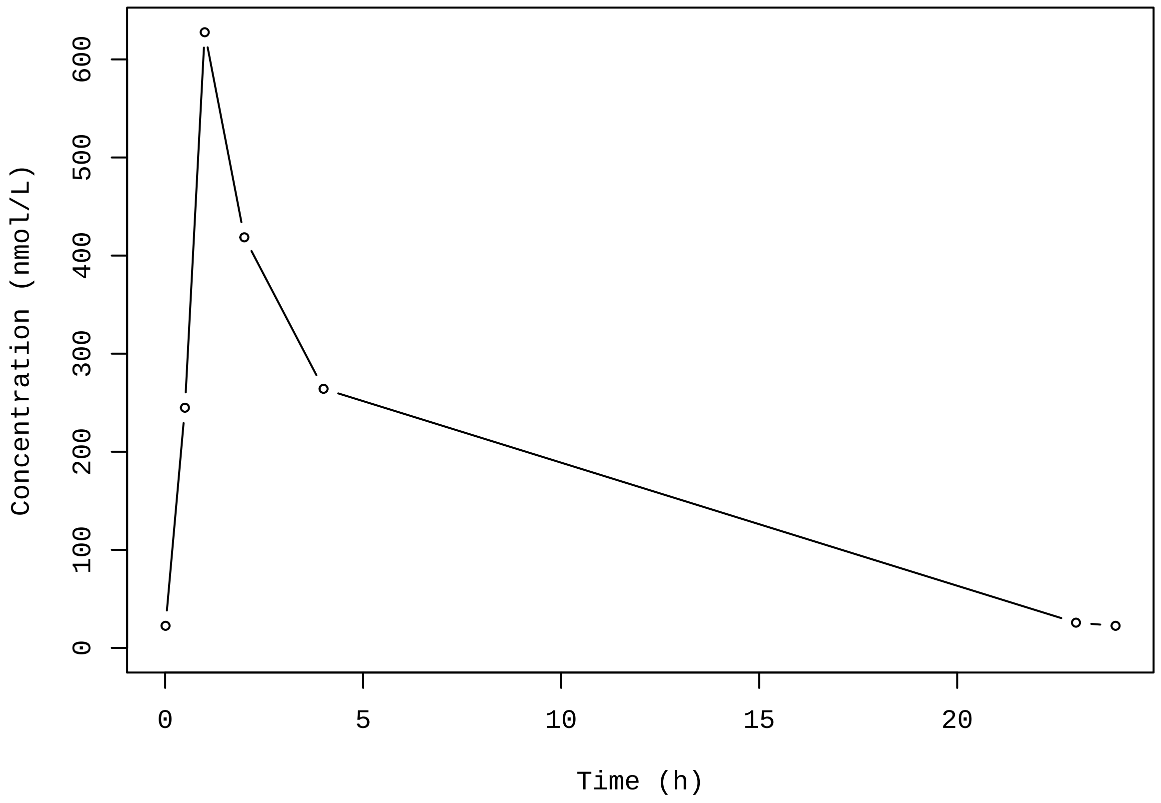

Concentration (nmol/L)
0
100
200
300
400
500
600
0
5
10
15
20
Time (h)

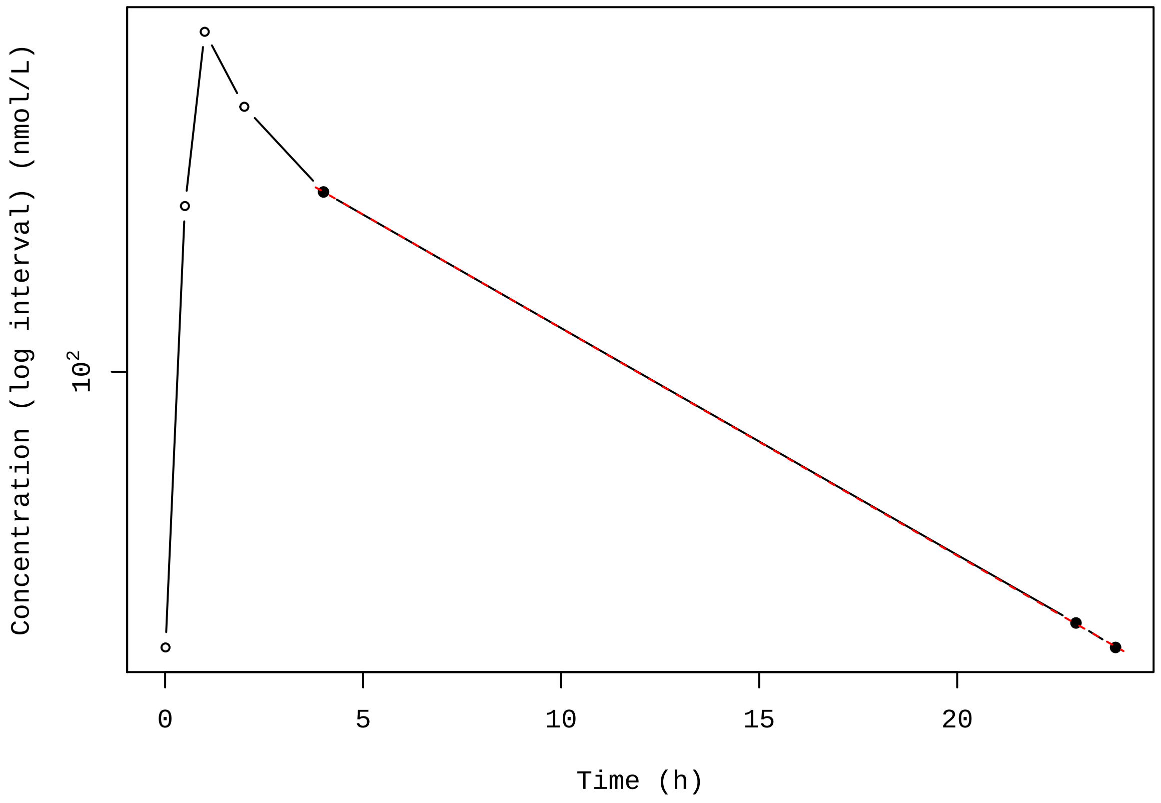

Concentration (log interval) (nmol/L)
10^2
0
5
10
15
20
Time (h)

Individual Noncompartmental Analysis Result

subj=3

NONCOMPARTMENTAL ANALYSIS REPORT
Package version 0.4.5 (2022−02−23)
R version 4.0.5 (2021−03−31)

Date and Time: 2022−07−12 23:15:03 Europe/Prague

Calculation Setting
−−−−−−−−−−−−−−−−−−−
Drug Administration: Extravascular
Observation count excluding trailing zero: 7
Dose at time 0: 0 mmol
AUC Calculation Method: Linear−up Linear−down
Weighting for lambda z: Uniform (Ordinary Least Square, OLS)
Lambda z selection criterion: Heighest adjusted R−squared value with precision=1e−4

Fitting, AUC, AUMC Result
−−−−−−−−−−−−−−−−−−−−−−−−−

Time		Conc.	Pred.	Residual	AUC	AUMC
0.0000	+	0.0000			0.0000	0.0000
0.0100		2.1000			0.0105	0.0001
0.5000		61.7000			15.6415	7.5635
1.0000		163.2000			71.8665	56.0760
2.0000		128.2000			217.5665	265.8760
4.0000	*	23.2000	23.1823	+1.774e−02	368.9665	615.0760
23.0000	*	2.3000	2.3355	−3.545e−02	611.2165	1999.2260
24.0000	*	2.1000	2.0697	+3.029e−02	613.4165	2050.8760

+: Back extrapolated concentration
*: Used for the calculation of Lambda z.

Calculated Values
−−−−−−−−−−−−−−−−−

CMAX	Max Conc	163.2000	nmol/L
TMAX	Time of CMAX	1.0000	h
TLAG	Time Until First Nonzero Conc	0.0000	h
CLST	Last Nonzero Conc	2.1000	nmol/L
CLSTP	Last Nonzero Conc Pred	2.0697	nmol/L
TLST	Time of Last Nonzero Conc	24.0000	h
LAMZHL	Half−Life Lambda z	5.7380	h
LAMZ	Lambda z	0.1208	/h
LAMZLL	Lambda z Lower Limit	4.0000	h
LAMZUL	Lambda z Upper Limit	24.0000	h
LAMZNPT	Number of Points for Lambda z	3	
CORRXY	Correlation Between TimeX and Log ConcY	−0.9999	
R2	R Squared	0.9999	
R2ADJ	R Squared Adjusted	0.9998	
AUCLST	AUC to Last Nonzero Conc	613.4165	h*nmol/L
AUCALL	AUC All	613.4165	h*nmol/L
AUCIFO	AUC Infinity Obs	630.8007	h*nmol/L
AUCIFP	AUC Infinity Pred	630.5500	h*nmol/L
AUCPEO	AUC %Extrapolation Obs	2.7559	%

subj=3

AUCPEP	AUC %Extrapolation Pred	2.7172	%
AUMCLST	AUMC to Last Nonzero Conc	2050.8760	h2*nmol/L
AUMCIFO	AUMC Infinity Obs	2612.0081	h2*nmol/L
AUMCIFP	AUMC Infinity Pred	2603.9131	h2*nmol/L
AUMCPEO	AUMC %Extrapolation Obs	21.4828	%
AUMCPEP	AUMC % Extrapolation Pred	21.2387	%
VZFO	Vz Obs by F	0.0000	L
VZFP	Vz Pred by F	0.0000	L
CLFO	Total CL Obs by F	0.0000	L/h
CLFP	Total CL Pred by F	0.0000	L/h
MRTEVLST	MRT Extravasc to Last Nonzero Conc	3.3434	h
MRTEVIFO	MRT Extravasc Infinity Obs	4.1408	h
MRTEVIFP	MRT Extravasc Infinity Pred	4.1296	h

subj=3

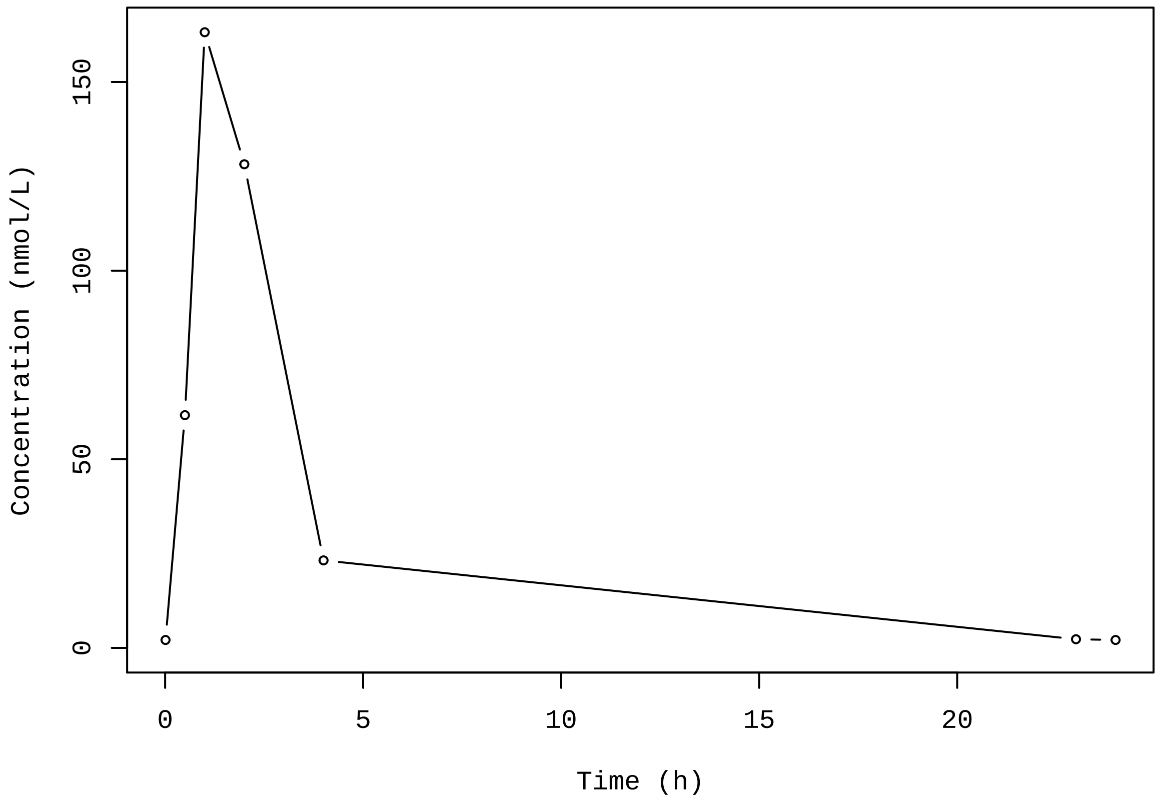

Concentration (nmol/L)
0
50
100
150
0
5
10
15
20
Time (h)

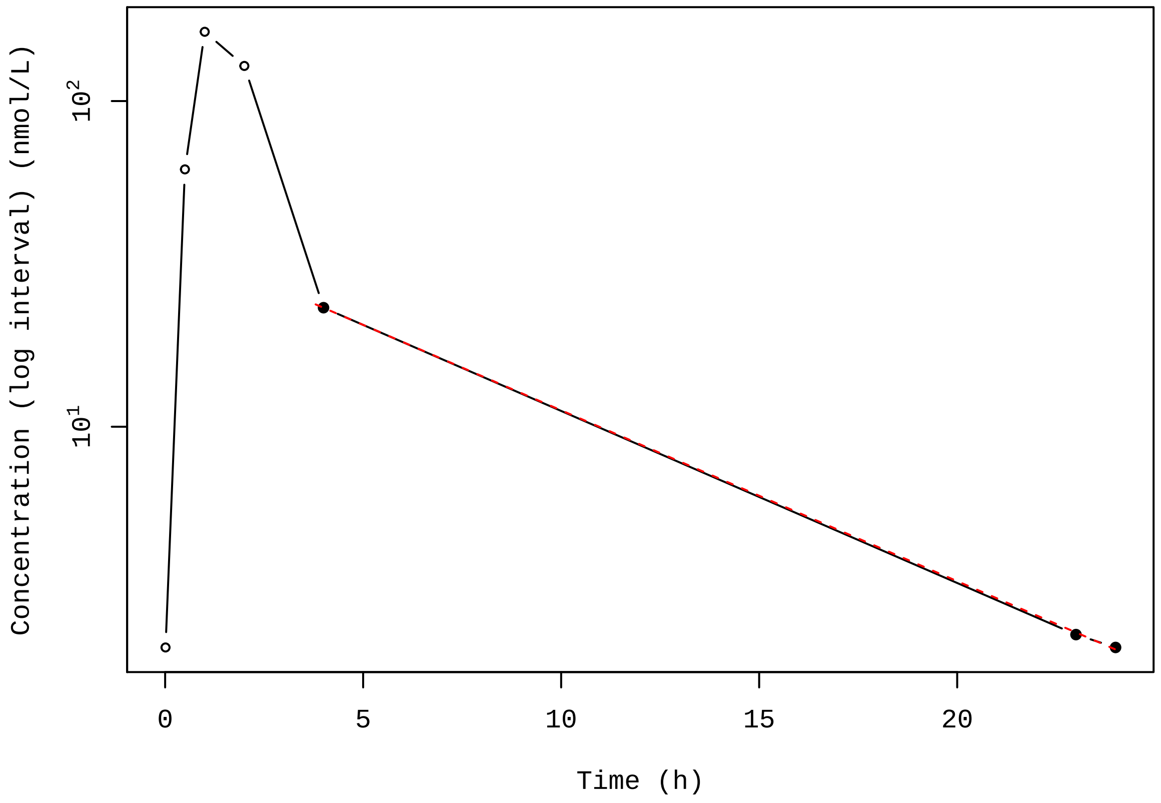

Concentration (log interval) (nmol/L)
10^1
10^2
0
5
10
15
20
Time (h)

Individual Noncompartmental Analysis Result

subj=3

NONCOMPARTMENTAL ANALYSIS REPORT
Package version 0.4.5 (2022−02−23)
R version 4.0.5 (2021−03−31)

Date and Time: 2022−07−12 22:35:21 Europe/Prague

Calculation Setting

Drug Administration: Extravascular
Observation count excluding trailing zero: 7
Dose at time 0: 0 mmol
AUC Calculation Method: Linear−up Linear−down
Weighting for lambda z: Uniform (Ordinary Least Square, OLS)
Lambda z selection criterion: Heighest adjusted R−squared value with precision=1e−4

Fitting, AUC, AUMC Result

Time		Conc.	Pred.	Residual	AUC	AUMC
0.0000	+	0.0000			0.0000	0.0000
0.0100		10.0948			0.0505	0.0005
0.5000		50.4412			14.8818	6.2043
1.0000		195.0797			76.2620	61.2794
2.0000		216.6386			282.1212	375.4578
4.0000	*	113.1746	113.0219	+1.527e−01	611.9343	1261.4332
23.0000	*	10.8205	11.1166	−2.961e−01	1789.8876	7926.3489
24.0000	*	10.0948	9.8392	+2.556e−01	1800.3453	8171.9228

+: Back extrapolated concentration
*: Used for the calculation of Lambda z.

Calculated Values

CMAX	Max Conc	216.6386	nmol/L
TMAX	Time of CMAX	2.0000	h
TLAG	Time Until First Nonzero Conc	0.0000	h
CLST	Last Nonzero Conc	10.0948	nmol/L
CLSTP	Last Nonzero Conc Pred	9.8392	nmol/L
TLST	Time of Last Nonzero Conc	24.0000	h
LAMZHL	Half−Life Lambda z	5.6787	h
LAMZ	Lambda z	0.1221	/h
LAMZLL	Lambda z Lower Limit	4.0000	h
LAMZUL	Lambda z Upper Limit	24.0000	h
LAMZNPT	Number of Points for Lambda z	3	
CORRXY	Correlation Between TimeX and Log ConcY	−0.9998	
R2	R Squared	0.9996	
R2ADJ	R Squared Adjusted	0.9993	
AUCLST	AUC to Last Nonzero Conc	1800.3453	h*nmol/L
AUCALL	AUC All	1800.3453	h*nmol/L
AUCIFO	AUC Infinity Obs	1883.0491	h*nmol/L
AUCIFP	AUC Infinity Pred	1880.9550	h*nmol/L
AUCPEO	AUC %Extrapolation Obs	4.3920	%

subj=3

AUCPEP	AUC %Extrapolation Pred	4.2856	%
AUMCLST	AUMC to Last Nonzero Conc	8171.9228	h2*nmol/L
AUMCIFO	AUMC Infinity Obs	10834.3795	h2*nmol/L
AUMCIFP	AUMC Infinity Pred	10766.9678	h2*nmol/L
AUMCPEO	AUMC %Extrapolation Obs	24.5741	%
AUMCPEP	AUMC % Extrapolation Pred	24.1019	%
VZFO	Vz Obs by F	0.0000	L
VZFP	Vz Pred by F	0.0000	L
CLFO	Total CL Obs by F	0.0000	L/h
CLFP	Total CL Pred by F	0.0000	L/h
MRTEVLST	MRT Extravasc to Last Nonzero Conc	4.5391	h
MRTEVIFO	MRT Extravasc Infinity Obs	5.7536	h
MRTEVIFP	MRT Extravasc Infinity Pred	5.7242	h

subj=3

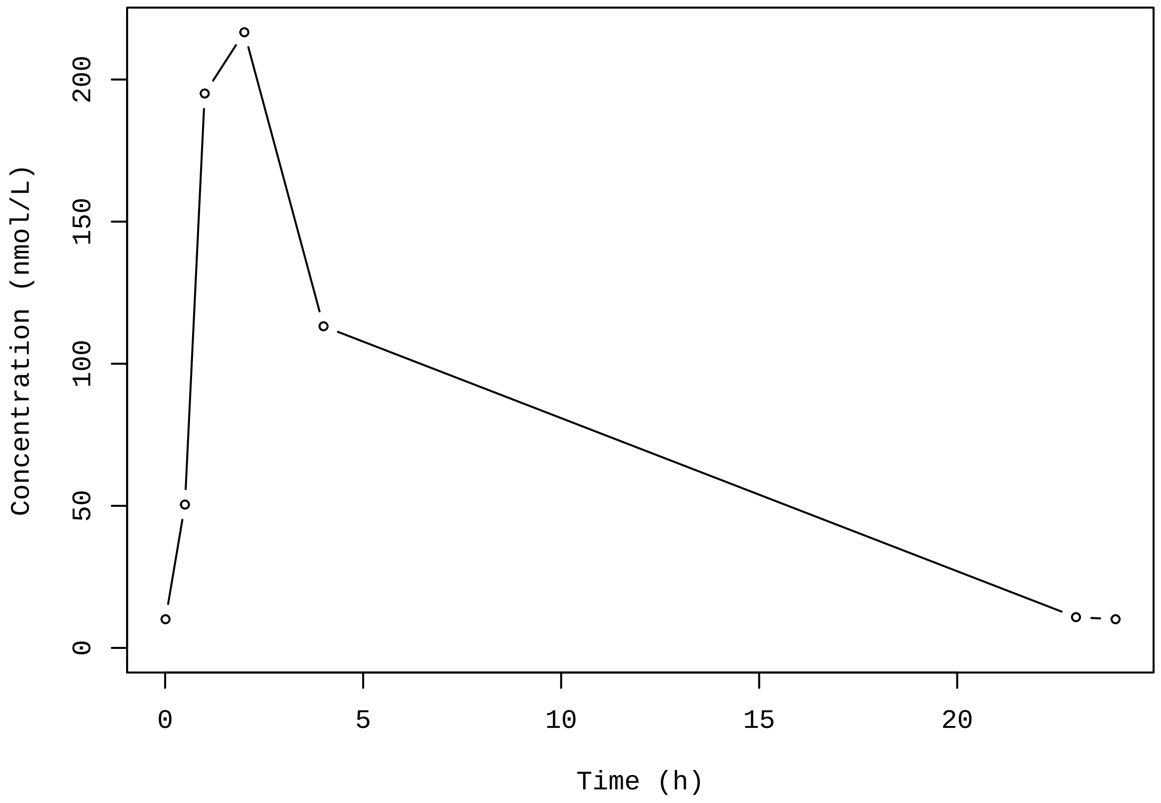

Concentration (nmol/L)
0
50
100
150
200
0
5
10
15
20
Time (h)

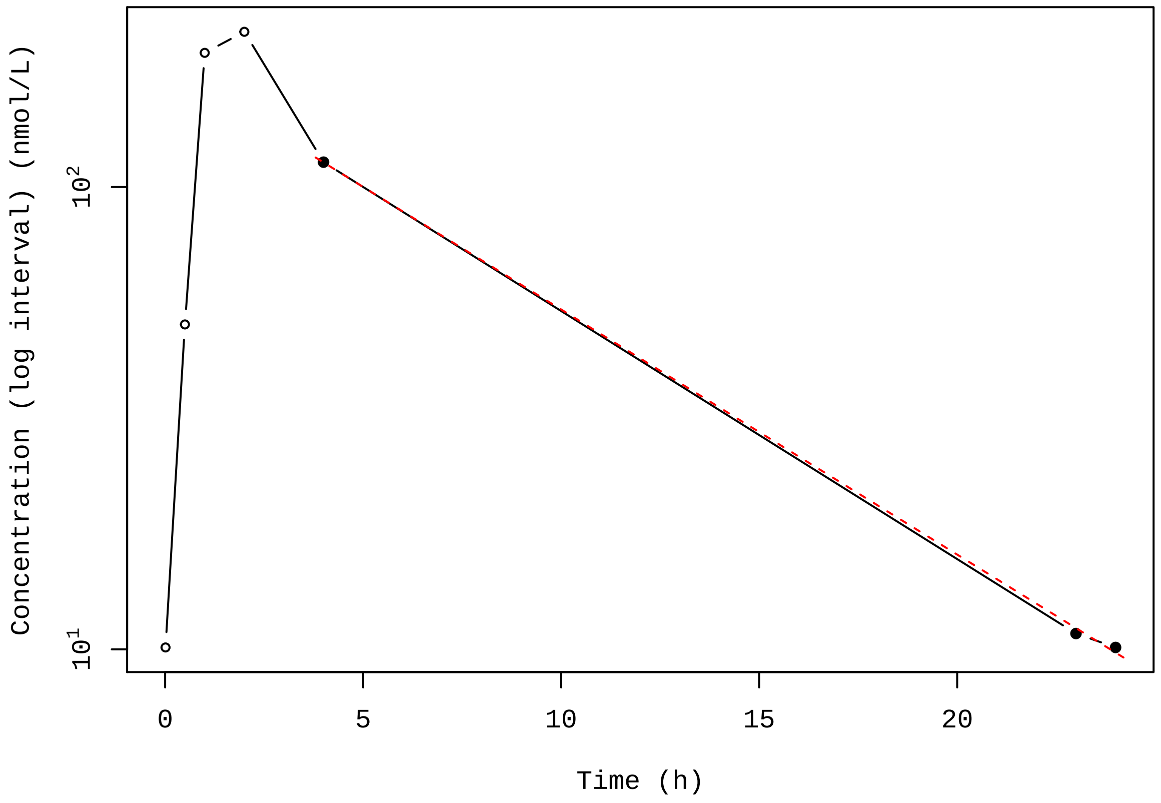

Concentration (log interval) (nmol/L)
10^1
10^2
0
5
10
15
20
Time (h)

Individual Noncompartmental Analysis Result

subj=3

NONCOMPARTMENTAL ANALYSIS REPORT
Package version 0.4.5 (2022−02−23)
R version 4.0.5 (2021−03−31)

Date and Time: 2022−04−02 23:24:21 Europe/Prague

Calculation Setting

Drug Administration: Extravascular
Observation count excluding trailing zero: 7
Dose at time 0: 0.636 mmol
AUC Calculation Method: Linear−up Linear−down
Weighting for lambda z: Uniform (Ordinary Least Square, OLS)
Lambda z selection criterion: Heighest adjusted R−squared value with precision=1e−4

Fitting, AUC, AUMC Result

Time		Conc.	Pred.	Residual	AUC	AUMC
0.0000	+	0.0000			0.0000	0.0000
0.0100		12.2197			0.0611	0.0006
0.5000		112.1059			30.5209	13.7635
1.0000		358.2700			148.1149	117.3443
2.0000		344.8633			499.6816	641.3426
4.0000	*	136.3376	136.1707	+1.669e−01	980.8825	1876.4198
23.0000	*	13.1573	13.4836	−3.264e−01	2401.0840	9932.1096
24.0000	*	12.2197	11.9385	+2.812e−01	2413.7725	10230.0544

+: Back extrapolated concentration
*: Used for the calculation of Lambda z.

Calculated Values

CMAX	Max Conc	358.2700	nmol/L
CMAXD	Max Conc Norm by Dose	563.3177	nmol/L/mmol
TMAX	Time of CMAX	1.0000	h
TLAG	Time Until First Nonzero Conc	0.0000	h
CLST	Last Nonzero Conc	12.2197	nmol/L
CLSTP	Last Nonzero Conc Pred	11.9385	nmol/L
TLST	Time of Last Nonzero Conc	24.0000	h
LAMZHL	Half−Life Lambda z	5.6952	h
LAMZ	Lambda z	0.1217	/h
LAMZLL	Lambda z Lower Limit	4.0000	h
LAMZUL	Lambda z Upper Limit	24.0000	h
LAMZNPT	Number of Points for Lambda z	3	
CORRXY	Correlation Between TimeX and Log ConcY	−0.9998	
R2	R Squared	0.9997	
R2ADJ	R Squared Adjusted	0.9994	
AUCLST	AUC to Last Nonzero Conc	2413.7725	h*nmol/L
AUCALL	AUC All	2413.7725	h*nmol/L
AUCIFO	AUC Infinity Obs	2514.1751	h*nmol/L
AUCIFOD	AUC Infinity Obs Norm by Dose	3953.1055	h*nmol/L/mmol

subj=3

AUCIFP	AUC Infinity Pred	2511.8648	h*nmol/L
AUCIFPD	AUC Infinity Pred Norm by Dose	3949.4730	h*nmol/L/mmol
AUCPEO	AUC %Extrapolation Obs	3.9935	%
AUCPEP	AUC %Extrapolation Pred	3.9052	%
AUMCLST	AUMC to Last Nonzero Conc	10230.0544	h2*nmol/L
AUMCIFO	AUMC Infinity Obs	13464.6704	h2*nmol/L
AUMCIFP	AUMC Infinity Pred	13390.2419	h2*nmol/L
AUMCPEO	AUMC %Extrapolation Obs	24.0230	%
AUMCPEP	AUMC % Extrapolation Pred	23.6007	%
VZFO	Vz Obs by F	2078.4817	L
VZFP	Vz Pred by F	2080.3933	L
CLFO	Total CL Obs by F	252.9657	L/h
CLFP	Total CL Pred by F	253.1983	L/h
MRTEVLST	MRT Extravasc to Last Nonzero Conc	4.2382	h
MRTEVIFO	MRT Extravasc Infinity Obs	5.3555	h
MRTEVIFP	MRT Extravasc Infinity Pred	5.3308	h

subj=3

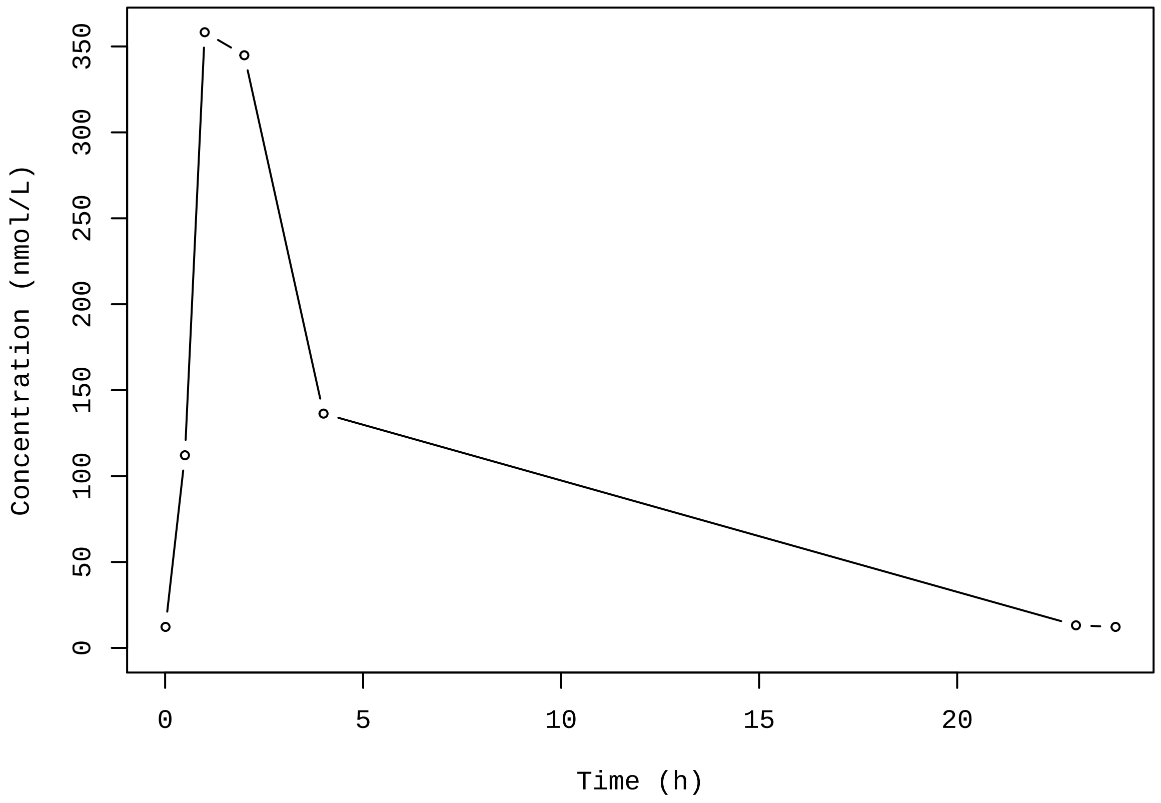

Concentration (nmol/L)
0
50
100
150
200
250
300
350
0
5
10
15
20
Time (h)

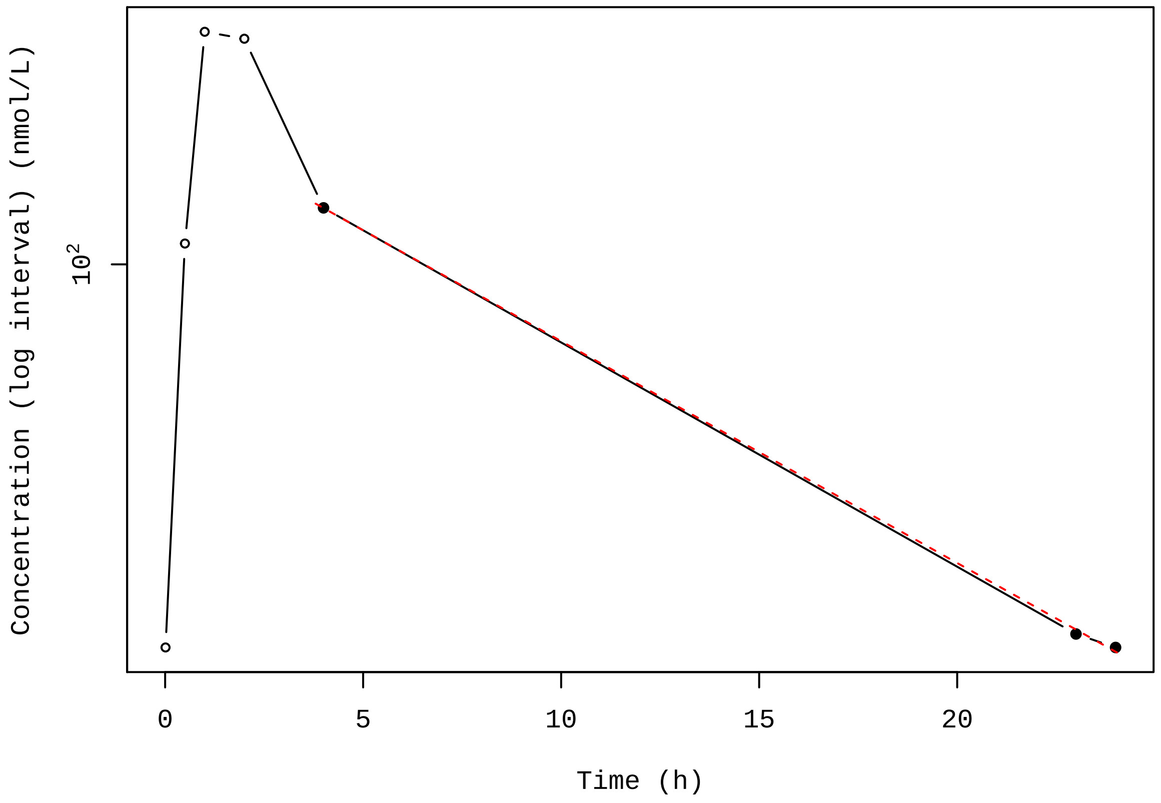

Concentration (log interval) (nmol/L)
10^2
0
5
10
15
20
Time (h)
